# CLEC18A Impairs Phagocytosis by Reducing FcγRIIA Expression and Arresting Autophagosome-Lysosome Fusion

**DOI:** 10.1128/spectrum.02903-22

**Published:** 2023-05-08

**Authors:** Tsai-Ling Liao, Yi-Ming Chen, Kuo-Tung Tang, Ying-Ying Yang, Der-Yuan Chen, Tsung-Hsien Chan, Hui-Ju Tsai, Shie-Liang Hsieh

**Affiliations:** a Department of Medical Research, Taichung Veterans General Hospital, Taichung, Taiwan; b Ph.D. Program in Translational Medicine, National Chung Hsing University, Taichung, Taiwan; c Rong Hsing Research Center for Translational Medicine, National Chung Hsing University, Taichung, Taiwan; d Division of Allergy, Immunology and Rheumatology, Taichung Veterans General Hospital, Taichung, Taiwan; e Division of Gastroenterology and Hepatology, Taipei Veterans General Hospital, Taipei, Taiwan; f Rheumatology and Immunology Center, China Medical University Hospital, Taichung, Taiwan; g Translational Medicine Laboratory, Rheumatology and Immunology Center, China Medical University Hospital, Taichung, Taiwan; h College of Medicine, China Medical University, Taichung, Taiwan; i Institute of Medicine, Chung Shan Medical University Hospital, Taichung, Taiwan; j Genomics Research Center, Academia Sinica, Taipei, Taiwan; k Immunology Research Center, National Health Research Institutes, Zhunan, Miaoli, Taiwan; l Institute of Clinical Medicine, National Yang Ming Chiao Tung University, Taipei, Taiwan; m Department of Medical Research, Taipei Veterans General Hospital, Taipei, Taiwan; National Center for Biological Sciences

**Keywords:** C-type lectin 18A, hepatitis C virus, mixed cryoglobulinemia, FcγRIIA, Rab7

## Abstract

Mixed cryoglobulinemia (MC) is a hepatitis C virus (HCV)–related extrahepatic manifestation that is characterized by the abnormal presence of immune complexes (ICs). This may be due to the reduced uptake and clearance of ICs. The C-type lectin member 18A (CLEC18A) is a secretory protein that is expressed abundantly in hepatocytes. We previously observed that CLEC18A increased significantly in the phagocytes and sera of patients with HCV, particularly those with MC. Herein, we explored the biological functions of CLEC18A in the MC syndrome development of patients with HCV by using an *in vitro* cell-based assay with quantitative reverse transcription-PCR, immunoblotting, immunofluorescence, flow cytometry, and enzyme-linked immunosorbent assays. HCV infection or Toll-like receptor 3/7/8 activation could induce CLEC18A expression in Huh7.5 cells. Upregulated CLEC18A interacts with Rab5 and Rab7 and enhances type I/III interferon production to inhibit HCV replication in hepatocytes. However, overexpressed CLEC18A suppressed phagocytic activity in phagocytes. Significantly decreased levels of the Fc gamma receptor (FcγR) IIA were found in the neutrophils of HCV patients, particularly in those with MC (*P *< 0.005). We demonstrated that CLEC18A could inhibit FcγRIIA expression in a dose-dependent manner through the production of NOX-2-dependent reactive oxygen species to impair the uptake of ICs. Additionally, CLEC18A suppresses the Rab7 expression that is induced by starvation. Overexpressed CLEC18A does not affect autophagosome formation but does reduce the recruitment of Rab7 to autophagosomes, thereby retarding the maturation of autophagosomes and affecting autophagosome-lysosome fusion. We offer a novel molecular machinery with which to understand the association of HCV infection with autoimmunity and propose that CLEC18A may act as a candidate biomarker for HCV-associated MC.

**IMPORTANCE** During infection, the host immune system produces cellular factors to protect against pathogen invasion. However, when the immune response overreacts and there is dysregulated cytokine homeostasis, autoimmunity occurs following an infection. We identified a cellular factor that is involved in HCV-related extrahepatic manifestation, namely, CLEC18A, which is expressed abundantly in hepatocytes and phagocytes. It inhibits HCV replication in hepatocytes by interacting with Rab5/7 and enhancing type I/III IFN expression. However, overexpressed CLEC18A inhibited FcγRIIA expression in phagocytes to impair phagocytosis. Furthermore, the interaction between CLEC18A and Rab5/7 may reduce the recruitment of Rab7 to autophagosomes and thereby retard autophagosome maturation and cause immune complex accumulation. A decreasing trend in CLEC18A levels that was accompanied by reduced HCV RNA titers and diminished cryoglobulin was observed in the sera of HCV-MC patients after direct-acting antiviral therapy. CLEC18A may be used for the evaluation of anti-HCV therapeutic drug effects and could be a potential predisposing factor for the development of MC syndrome.

## INTRODUCTION

Hepatitis C virus (HCV) infection is a major health problem (1). In addition to liver damage, numerous HCV extrahepatic manifestations (HCV-EHMs) have been reported among patients with chronic HCV infections (2, 3). The molecular machinery accounting for the association between HCV infection and autoimmunity is still uncertain. Mixed cryoglobulinemia (MC) is the most common HCV-EHM, and it is characterized by the formation of circulating immune complexes (ICs), called cold-precipitable cryoglobulin complexes, that are composed of immunoglobulin antibodies (monoclonal IgM and polyclonal IgG) with rheumatoid factor activity (4–6). In addition to antibodies, HCV particles and nonenveloped nucleocapsid proteins are involved in the formation of cryoglobulins (6). Fc gamma receptors (FcγRs) can trigger the internalization of captured ICs, which leads to the degradation of antigen-antibody complexes and plays a crucial role in the clearance of ICs (7). Accumulating evidence has suggested that FcγRs may be associated with MC pathogenesis (8), but the regulatory mechanism is still unclear.

The C-type lectin member 18A (CLEC18A) belongs to the CLEC family, which is expressed abundantly in normal hepatocytes and peripheral blood cells (9). CLEC18A encodes a 448 amino acid polypeptide with a typical C-type lectin domain that contains the typical signatures “WIGL” (aa 370 to 373), “QPD” (aa 399 to 401), and “WND” (aa 419 to 421). Sugar binding assays demonstrated that CLEC18A binding to F3 polysaccharides was inhibited efficiently by polysaccharides (such as laminarin, pachyman, fucoidan, and galatan). Unlike DC-SIGN, CLEC18A binding is not inhibited by monosaccharides (such as GluNac, galactose, mannose, and fucose) (9). This observation suggests that CLEC18A prefers binding to sulfated fucose, glucan, and galactan. It is localized in the endoplasmic reticulum, Golgi apparatus, and endosome (9). We have demonstrated that CLEC18A is a Toll-like receptor (TLR) 3 coreceptor and may contribute to host immune responses to viral infection (10). Tsai et al. (11) showed that plasma CLEC18A levels are correlated with the stage of hepatitis B virus infection and could be a potential biomarker with which to predict the outcomes of patients. We previously observed that CLEC18A is significantly associated with HCV infection, particularly HCV-associated MC (12). We found that CLEC18A is highly expressed in the phagocytes (e.g., monocytes and neutrophils) of patients with HCV-associated MC (12). We also found a positive correlation between CLEC18A expression and cryoglobulin levels (*r *= 0.43, *P *< 0.05), which suggests that CLEC18A may be involved in the formation of ICs in MC pathogenesis. In this study, we explored the biological function of CLEC18A in HCV infection and investigated its role in the molecular mechanisms of HCV-associated MC development. We analyzed the expression of FcγR autophagy-related genes (LC3, ATG5, and p62) in the peripheral blood cells of patients with HCV-associated MC, compared with patients with HCV without MC or healthy controls by using quantitative reverse transcription PCR (qRT-PCR). We also investigated the role of CLEC18A in phagocytosis by using immunoblotting, immunofluorescence, flow cytometry, and functional phagocytosis assays.

## RESULTS

### Increased CLEC18A expression in hepatocytes with HCV infection following TLR3/7/8 ligand stimulation.

**FIG 1:**
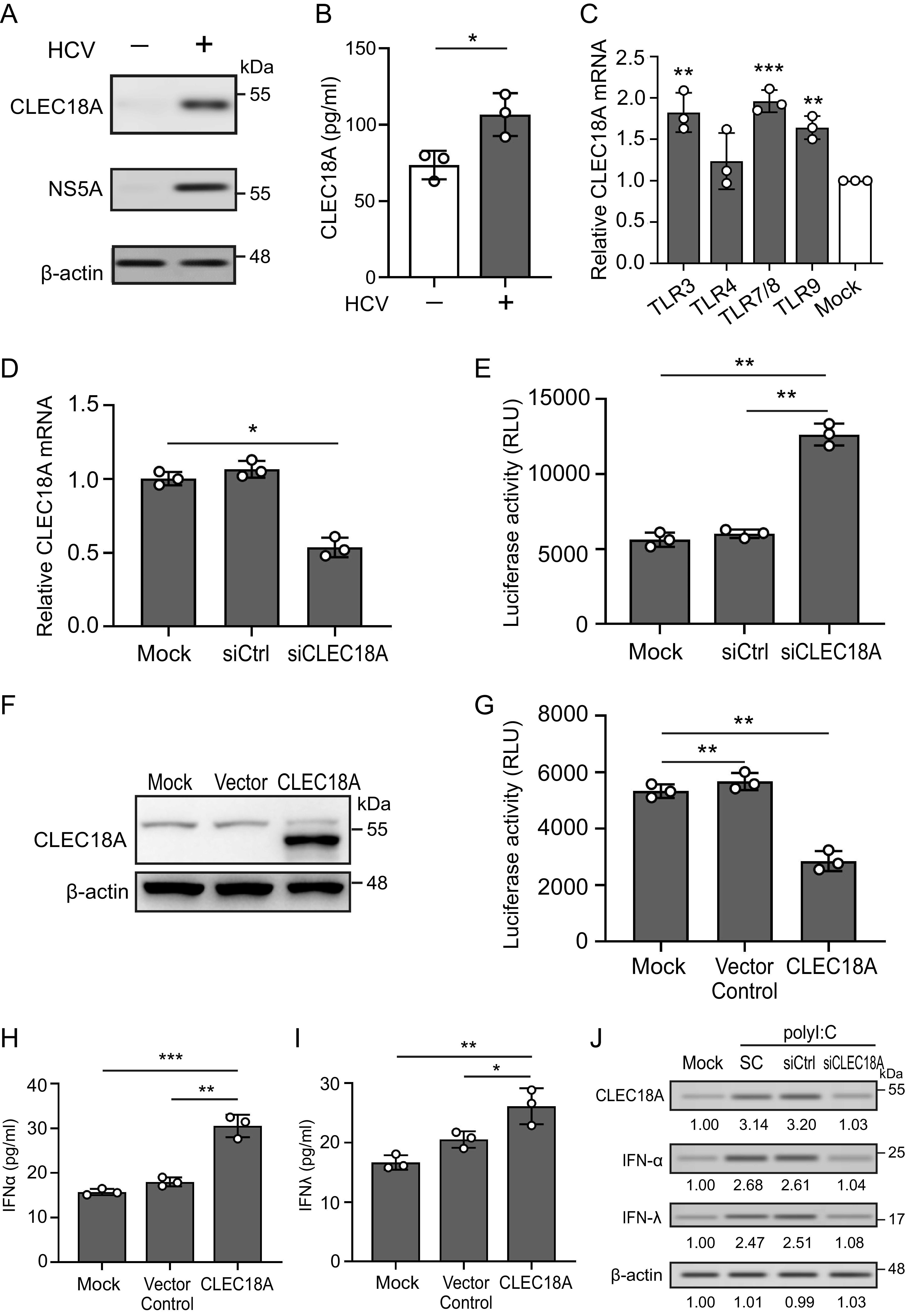
Increased CLEC18A expression in hepatocytes with HCV infection to inhibit viral replication. (A and B) Huh7.5 cells were infected with the HCVcc JC1 strain at a MOI of 0.1. At 72 hours postinfection, the levels of (A) intracellular and (B) secretory CLEC18A were analyzed. (C) The expression of CLEC18A in Huh7.5 cells after the stimulation of different TLR ligands was analyzed by using qRT-PCR and normalized by using GAPDH. (D–G) HCV-tricistronic replicon cells were transfected with (D and E) siCLEC18A to knock down CLEC18A or (F and G) pCMV-CLEC18A to overexpress CLEC18A. After 72 h, the expression of CLEC18A was detected via qRT-PCR or immunoblotting, and HCV RNA replication activity was measured by using a luciferase assay. (H and I) Huh7 cells were transfected with pCMV-CLEC18A to overexpress CLEC18A. After 24 h, the cells were transfected with the TLR3 ligand polyriboinosinic:polyribocytidylic acid (poly [I·C]). The supernatant was collected, and the levels of (H) IFN-α and (I) IFN-λ were measured. (J) CLEC18A knockdown cells were transfected with poly (I·C). The levels of CLEC18A, IFN-α, IFN-λ, and β-actin were detected via immunoblotting. The immunoblotting bands from β-actin were densitometrically measured using ImageJ to determine the lane normalization factor for the samples. All of the experiments were performed in triplicate, and the data are presented as the mean ± SD. The image shown is from a single experiment that is representative of at least three separate experiments. The results of the densitometric analysis were presented in in Fig. S8 in the supplemental material. *, *P *< 0.05; **, *P *< 0.01; ***, *P *< 0.005.

To investigate whether CLEC18A expression is associated with HCV infection, Huh7.5 cells were infected with the HCVcc JC1 strain at a multiplicity of infection (MOI) of 0.1 for 72 h. At 72 hours postinfection, the HCV-infected cells were collected for CLEC18A expression analysis via immunoblotting. There was increased intracellular and secretory CLEC18A expression in the HCV-infected cells, compared with the uninfected cells (Fig. 1A and B). We further investigated whether innate immunity-associated TLRs might regulate CLEC18A expression in hepatocytes. There was a significant increase in CLEC18A in Huh7.5 cells after treatment with the TLR3 ligand polyriboinosinic:polyribocytidylic acid (poly [I·C]) (1.82 ± 0.24-fold, *P *< 0.01) (Fig. 1C), the TLR7/8 ligand resiquimod (R848) (1.96 ± 0.13-fold, *P *< 0.005), or the TLR9 ligand CpG (1.64 ± 0.14-fold, *P *< 0.01). These changes suggest that double-stranded RNA, single-stranded RNA, or double-stranded DNA might contribute to CLEC18A upregulation in hepatocytes. A slightly higher level of CLEC18A was observed in cells after TLR4 ligand stimulation (1.24 ± 0.20-fold), but this result did not reach statistical significance.

### CLEC18A inhibits HCV replication in hepatocytes.

To determine the biological function of CLEC18A in HCV infection, we tested the replication activity of HCV by using an HCV-tricistronic replicon cell system (13) that contained luciferase as the reporter and assays viral RNA replication activity. When cellular CLEC18A was knocked down (Fig. 1D), the HCV replication activity was enhanced by approximately 2.25-fold (Fig. 1E) (luciferase activity: 12,630.0 ± 729.4 versus 5,624.1 ± 476.7 RLU). Conversely, when CLEC18A was overexpressed in the HCV replicon cells (Fig. 1F), the HCV replication activity was reduced to about 0.54-fold (Fig. 1G) (2,851.3 ± 353.7 versus 5,330.0 ± 239.5 RLU).

### CLEC18A enhances the production of type I and type III IFNs in hepatocytes.

Type I and III IFNs play a key role in anti-HCV infection and are induced upon TLR3 stimulation. Recently, we demonstrated that CLEC18A is a TLR3 coreceptor (10). Hence, we assessed the effect of CLEC18A on the production of type I and type III IFNs in hepatocytes upon TLR3 activation. This activation elevated the levels of IFN-α (30.50 ± 2.54 pg/mL versus 15.70 ± 0.69 pg/mL, *P *< 0.005) (Fig. 1H) and IFN-λ (26.12 ± 3.01 pg/mL versus 16.66 ± 1.22 pg/mL, *P *< 0.01) (Fig. 1I) in CLEC18A-overexpressing cells, compared with control cells. Moreover, when CLEC18A was knocked down, the effect of TLR3 activation on the upregulation of IFNs was suppressed (Fig. 1J) (*P < *0.005).

The recent advent of direct-acting antivirals (DAAs) has achieved remarkable therapeutic efficacy in HCV patients (14). We analyzed the dynamics of the HCV titer, cryoglobulin concentration, and CLEC18A expression in the sera of HCV-MC patients receiving DAAs therapy. Our results showed that dramatically decreased HCV viral titers could be observed in patients after DAAs therapy (before versus after: 9.2 ± 5.8 ×10^5^ IU/mL versus undetectable, *P < *0.005) (Fig. S1A), and these were accompanied with declined cryoglobulin (158.0 ± 121.2 versus 0.03 ± 0.02 μg/mL, *P *< 0.05) (Fig. S1B) and reduced levels of CLEC18A (233.2 ± 67.9 versus 70.5 ± 13.3 pg/mL, *P *< 0.05) (Fig. S1C).

### CLEC18A inhibits phagocytosis.

**FIG 2:**
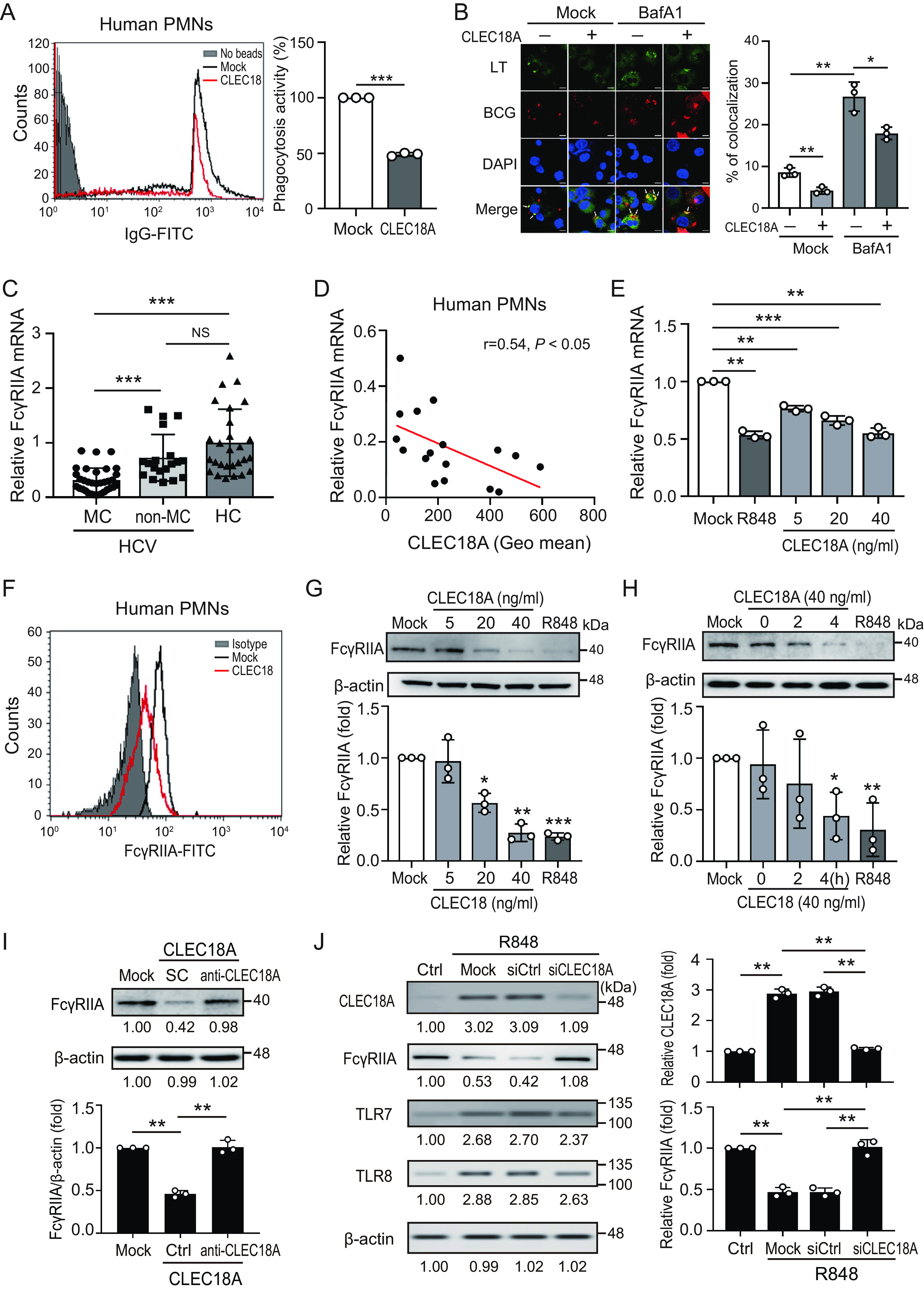
CLEC18A inhibits phagocytosis and FcγRIIA expression. (A) Human neutrophils were treated with CLEC18A (40 ng/mL) for 4 h. IgG-FITC beads were added at a ratio of 1:200. After 4 h, the phagocytic activity was analyzed and quantified by using flow cytometry. (B) THP-1 cell-derived macrophages were treated with or without CLEC18A (40 ng/mL) in the absence or presence of bafilomycin A1 (BafA1, 100 nM) for 24 h, and they were subsequently infected with Texas Red-labeled M. bovis BCG for 2 h and stained with LysoTracker Green (LT, 2 μM). LT-positive puncta (green) and BCG (red) were detected via confocal microscopy (left panel). The fraction (%) of mycobacterium-containing phagosomes colocalizing with LT puncta was quantified (right panel). The scale bar in the immunofluorescence assay (IFA) image represents 5 μm. (C) Decreased FcγRIIA mRNA in neutrophils from patients with HCV-associated MC, compared to those without MC or healthy controls (HC). (D) The expression of FcγRIIA was negatively correlated with the CLEC18A levels in neutrophils in patients with HCV-associated MC. (E) dHL60 cells were treated with the indicated concentrations of CLEC18A for 4 h. FcγRIIA mRNA was measured by using qRT-PCR. (F) Human neutrophils were treated with CLEC18A (40 ng/mL) for 4 h. The level of FcγRIIA was analyzed by using flow cytometry. (G and H) CLEC18A inhibits FcγRIIA expression in human neutrophils in a (G) dose-dependent and (H) time-dependent manner. (I) Human neutrophils were treated with CLEC18A (40 ng/mL) in the presence of anti-CLEC18A antibodies (10 μg/mL) for 4 h. PBS was used as a solvent control (SC). The levels of FcγRIIA were detected and quantified via an immunoblotting analysis. (J) CLEC18A knockdown or control cells were treated with R848 for 24 h, and the levels of CLEC18A, FcγRIIA, TLR7, and TLR8 were analyzed and quantified using immunoblotting. The immunoblotting bands from β-actin were densitometrically measured using ImageJ to determine the lane normalization factor for the samples. The image shown is from a single experiment that is representative of at least three separate experiments. The data are presented as the mean ± SD. *, *P *< 0.05; **, *P *< 0.01; ***, *P *< 0.005; NS, not significant.

MC is characterized by the accumulation of cryoglobulin ICs. Phagocytosis is a major pathway to clear ICs. We observed an increased level of CLEC18A in the phagocytes and sera of patients with HCV-associated MC (12). Given that HCV infection or Toll-like receptor 3/7/8 activation could induce CLEC18A expression but that mature neutrophils express all TLRs except TLR3 (15), we investigated the association between the TLR7/8-CLEC18A axis in phagocytes and the pathogenesis of HCV-MC. Increased CLEC18A expression was shown in human neutrophils after treatment with TLR7/8 ligand R848 (mean of fluorescence of intensity [MFI]: 240.0 ± 20.6 versus 167.5 ± 2.8, *P *< 0.05) (Fig. S2). We performed a phagocytosis assay to unravel the association between CLEC18A and phagocytosis. The phagocytic activity was suppressed in the presence of CLEC18A, compared with control cells (48.9% ± 1.5% versus 100%, *P *< 0.005) (Fig. 2A). We further evaluated the effect of CLEC18A on phagocytosis by using an immunofluorescence assay. THP-1-derived macrophages were treated with CLEC18A in the presence or absence of BafA1 for 24 h. LysoTracker (LT) is a fluorescent probe that is widely used for the viable cell staining of autophagosomes, phagosomes, autolysosomes, and lysosomes. The cells were infected with Texas Red-labeled Mycobacterium bovis bacillus Calmette-Guérin (BCG) at a MOI of 10, and the phagocytic activity was examined by calculating the ratio of LT-positive organelles containing BCG with confocal microscopy. There was a decreased ratio of the colocalization of BCG with LT-positive organelles in the CLEC18A-treated cells, compared with the control cells (4.2% ± 0.8% versus 8.6% ± 1.1%, *P *< 0.01) (Fig. 2B). In addition, BafA1 increased the ratio of the colocalization of BCG with LT-positive organelles (26.7% ± 3.5%, *P *< 0.01), which was reduced in the presence of CLEC18A (17.9% ± 1.5%, *P *< 0.05). Our results suggest that CLEC18A may inhibit phagocytosis.

### Upregulated CLEC18A inhibits FcγRIIA expression.

FcγRs are mainly on phagocytes and play a crucial role in phagocytosis for the clearance of ICs. To assess the association between the expression of FcγRs and the occurrence of HCV-associated MC symptoms, we compared the levels of FcγRIIA, FcγRIIB, FcγRIIIA, and FcγRIIIB in neutrophils from patients with or without HCV-associated MC by using qRT-PCR. There were no significant differences in the levels of FcγRIIB, FcγRIIIA, and FcγRIIIB in neutrophils from patients with or without HCV-associated MC (Fig. S3). FcγRIIA was significantly reduced in patients with HCV-associated MC (*n* = 33), compared with those without HCV-associated MC (*n* = 19) or healthy controls (HC, *n* = 27) (0.31 ± 0.23 versus 0.72 ± 0.43 versus 1.00 ± 0.61-fold, *P* < 0.001) (Fig. 2C). Moreover, there was a negative correlation between CLEC18A expression and FcγR2A levels in the neutrophils of patients with HCV-associated MC (*r* = 0.54, *P *< 0.05) (Fig. 2D). To explore the effect of CLEC18A on FcγRIIA expression, we measured the mRNA and protein levels of FcγRIIA in human neutrophils treated with CLEC18A. The results revealed that CLEC18A could inhibit FcγRIIA expression in a dose-dependent (40 ng/mL: 0.27 ± 0.09-fold, *P *< 0.01) (Fig. 2E–G) and time-dependent manner (4 h: 0.44 ± 0.23-fold, *P *< 0.05) (Fig. 2H). This effect was diminished in the presence of anti-CLEC18A antibodies (10 μg/mL) (*P *< 0.01) (Fig. 2I). Lood et al. (16) demonstrated that TLR7/8 activation in phagocytes impairs phagocytosis by shedding FcγRIIA. We further confirmed the association between the TLR7/8 activation-CLEC18A axis and FcγRIIA expression. Increased CLEC18A expression was shown in human neutrophils after treatment with R848, which is accompanied by decreased FcγRIIA expression (Fig. 2J). When intracellular CLEC18A was knocked down efficiently, R848-induced decreased FcγRIIA expression was rescued. Our results suggest that CLEC18A may be involved in TLR7/8 activation-associated FcγRIIA shedding.

**FIG 3:**
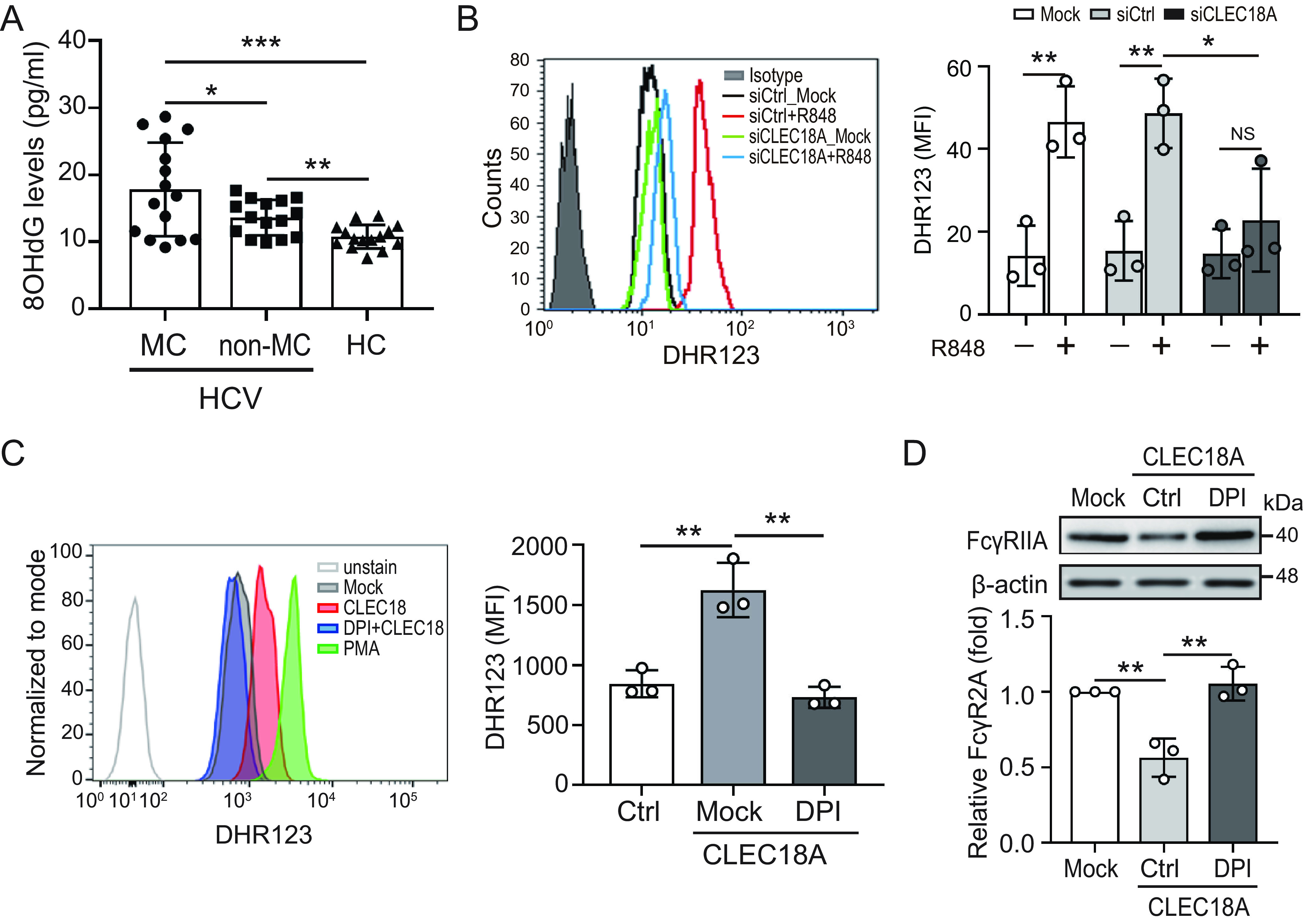
CLEC18A inhibited FcγRIIA expression by mediating NOX2-dependent ROS production. (A) Increased oxidative stress (8-OHdG) in patients with HCV-associated MC. (B) CLEC18A knockdown or control cells were treated with resiquimod (R848) for 24 h. The cytosolic ROS level was analyzed using dihydrorhodamine (DHR)123 dye with flow cytometry. (C) Human neutrophils were treated with the indicated reagent for 4 h. The level of cytosolic ROS was analyzed by using DHR123 with flow cytometry. (D) Human neutrophils were treated with CLEC18A (40 ng/mL) in the absence or presence of the NOX2 inhibitor diphenyleneiodonium (DPI, 25 μM) for 4 h. FcγRIIA expression was analyzed and quantified by using immunoblotting. The immunoblotting bands from β-actin were densitometrically measured using ImageJ to determine the lane normalization factor for the samples. The image shown is from a single experiment that is representative of at least three separate experiments. All of the experiments were performed in triplicate, and the data are presented as the mean ± SD. *, *P *< 0.05; **, *P *< 0.01; ***, *P *< 0.005; NS, not significant.

A previous study demonstrated that reactive oxygen species (ROS) causes the release of proteases to suppress FcγRIIA expression in immune cells (16). We observed that oxidative stress (8-OHdG) was higher in patients with HCV-associated MC (*n* = 15), compared with patients with HCV without MC (*n* = 15) or healthy controls (17.83 ± 6.99 versus 13.62 ± 2.64 versus 10.75 ± 1.77 pg/mL, *P* < 0.05) (Fig. 3A). These results suggest that ROS may be associated with MC pathogenesis. We further showed that R848 induces the production of cytosolic ROS (MFI: 46.55 ± 8.61 versus 14.21 ± 7.28, *P *< 0.01) (Fig. 3B), which is consistent with another report (16). In addition, the effect of R848 on the production of cytosolic ROS was suppressed when CLEC18A was knocked down (MFI: 22.81 ± 12.44), suggesting that CLEC18A may play a key role in R848-induced cytosolic ROS production.

In addition, we showed that recombinant CLEC18A treatment induced greater ROS production, compared with the control cells (MFI: 1,625.2 ± 226.4 versus 845.3 ± 113.2, *P *< 0.01) (Fig. 3C). Increased CLEC18A-induced ROS were inhibited in the presence of the nicotinamide-adenine dinucleotide phosphate oxidase (NOX) inhibitor diphenyleneiodonium (DPI, 25 μM) (MFI: 730.8 ± 88.0, *P *< 0.01) (Fig. 3C). We further explored the association between CLEC18A, FcγRIIA, and ROS by using immunoblotting (Fig. 3D). CLEC18A inhibited FcγRIIA expression (0.56 ± 0.13-fold, *P *< 0.01), which could be rescued in the presence of DPI (1.05 ± 0.11-fold, *P *< 0.01).

### CLEC18A inhibits autophagic flux.

**FIG 4:**
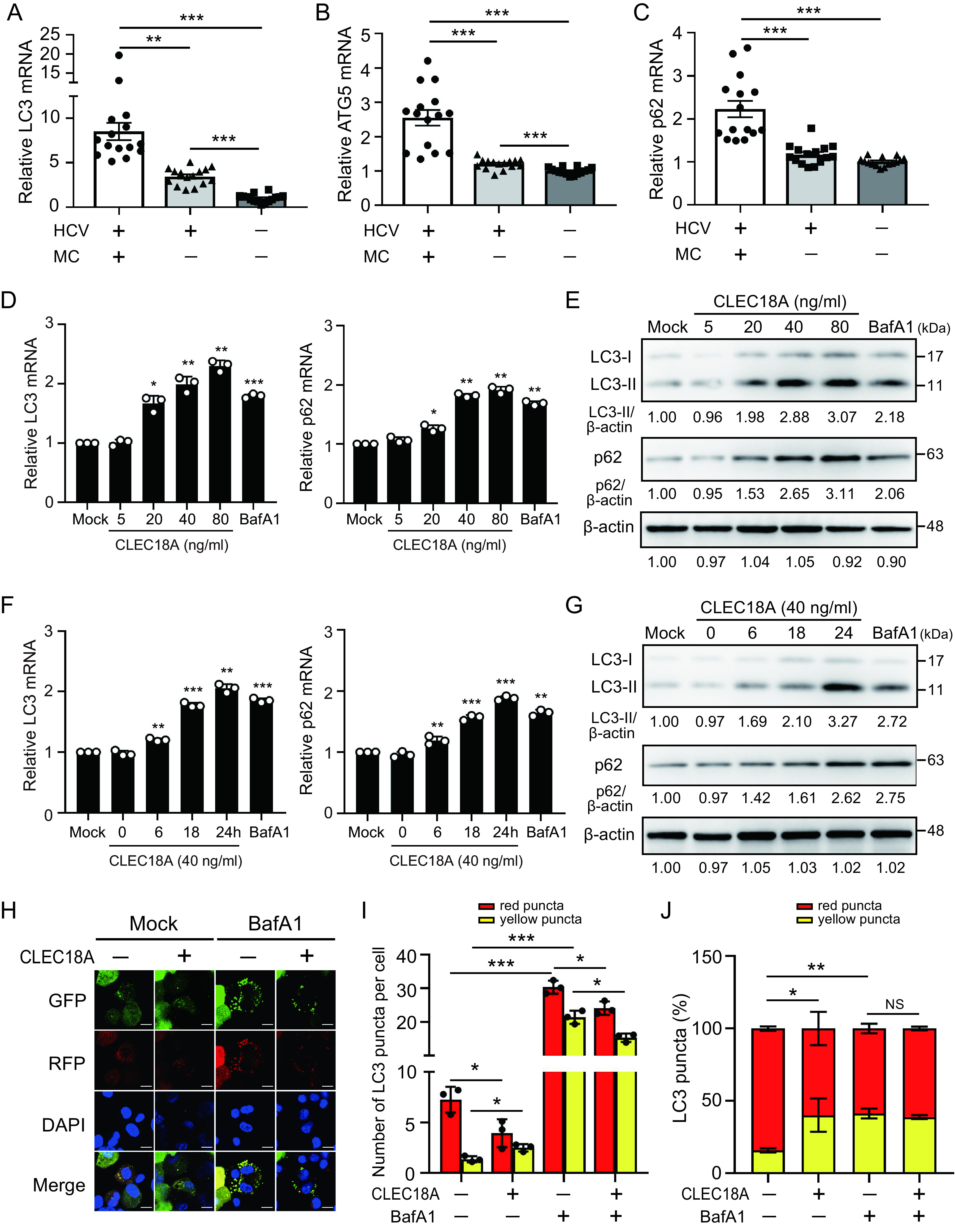
CLEC18A inhibits autophagic flux. (A and B) Increased expression of autophagy-related (A) LC3, (B) ATG5, and (C) p62 were detected in PBMCs from patients with HCV-associated MC symptoms. (D–G) THP-1-derived macrophages were treated with (D and E) the indicated concentrations of CLEC18A for 24 h or (F and G) CLEC18A (40 ng/mL) for the indicated time. The expression of LC3 and p62 was detected by using qRT-PCR and immunoblotting, respectively. The immunoblotting bands from β-actin were densitometrically measured using ImageJ to determine the lane normalization factor for the samples. The image shown is from a single experiment that is representative of at least three separate experiments. (H to J) THP-1 cells stably expressing the RFP-GFP-LC3 fusion protein were treated with the indicated reagent for 24 h and observed via (H) confocal microscopy. (I) The numbers of RFP^+^ GFP^−^ LC3 (red) and RFP^+^ GFP^+^ LC3 (yellow) puncta per cell in individual treatments were quantified. (J) The percentage of total of yellow puncta (autophagosomes) and red puncta (autolysosomes) per cell in individual treatments. All of the experiments were performed in triplicate, and the data are presented as the mean ± SD. The scale bar in the IFA image represents 10 μm. *, *P *< 0.05; **, *P* < 0.01; ***, *P *< 0.005; NS, not significant.

In addition to FcγRs, autophagy is another key factor responsible for ICs removal. To assess whether autophagy is impaired in MC, we analyzed the expression of autophagy-related genes (e.g., LC3, ATG5, and p62) in patients with MC by using qRT-PCR. The expression of LC3 (8.53 ± 3.74-fold, Fig. 4A), ATG5 (2.55 ± 0.88-fold, Fig. 4B), and p62 (2.23 ± 0.74-fold, Fig. 4C) increased in the peripheral blood mononuclear cells (PBMCs) of patients with HCV-associated MC (*n* = 15) compared with patients with HCV but not MC (*n* = 15, LC3: 3.46 ± 0.96-fold, *P < *0.01; ATG5: 1.21 ± 0.15-fold, *P < *0.005; p62: 1.17 ± 0.23-fold, *P < *0.005) or healthy controls (*n* = 15, LC3: 1.00 ± 0.47-fold, *P < *0.005; ATG5: 1.00 ± 0.12-fold, *P < *0.005; p62: 1.00 ± 0.10-fold, *P < *0.005). The results suggest that MC symptoms may be associated with impaired autophagic flux.

To evaluate the effect of CLEC18A on autophagic flux, THP-1-derived macrophages were treated with CLEC18A, and the level of LC3 or degradation of p62 was measured via qRT-PCR or immunoblotting, respectively. There was increased LC3-II and an accumulation of p62 after CLEC18A treatment in a dose-dependent (Fig. 4D and E) and time-dependent (Fig. 4F and G) manner.

We further examined the effect of CLEC18A on autophagic flux by using THP-1 cells that were stably expressing the RFP-GFP-LC3 fusion protein. CLEC18A induced a redistribution of the RFP-GFP-LC3 fusion protein from a diffused to a punctate pattern (Fig. 4H), which resulted in a decrease in the number of red puncta (3.9 ± 0.8 versus 7.3 ± 0.7, *P *< 0.05) (Fig. 4I) and an increase in the number of yellow puncta (2.5 ± 0.5 versus 1.4 ± 0.2, *P *< 0.05) (Fig. 4I), compared with the control cells. BafA1 is an autophagosome degradation inhibitor that regulates luminal acidification. An increased percentage of yellow puncta was revealed in cells after treating with BafA1 (41.3 ± 3.4%, *P *< 0.01) (Fig. 4J) or CLEC18A (40.2 ± 11.4%, *P *< 0.05), compared with those in control cells (15.8 ± 1.4%). Although there was a decrease in the number of BafA1-induced red puncta and yellow puncta after CLEC18A treatment (red puncta: 24.1 ± 1.1 versus 30.4 ± 1.1, *P *< 0.05; yellow puncta: 15.3 ± 0.7 versus 21.4 ± 1.1, *P *< 0.05) (Fig. 4I), there was no significant difference in the percentage of red/yellow puncta in BafA1-induced cells with or without CLEC18A treatment (Fig. 4J). Our results suggested that CLEC18A may suppress autophagic flux.

### CLEC18A suppressed the fusion of autophagosomes with lysosomes.

**FIG 5:**
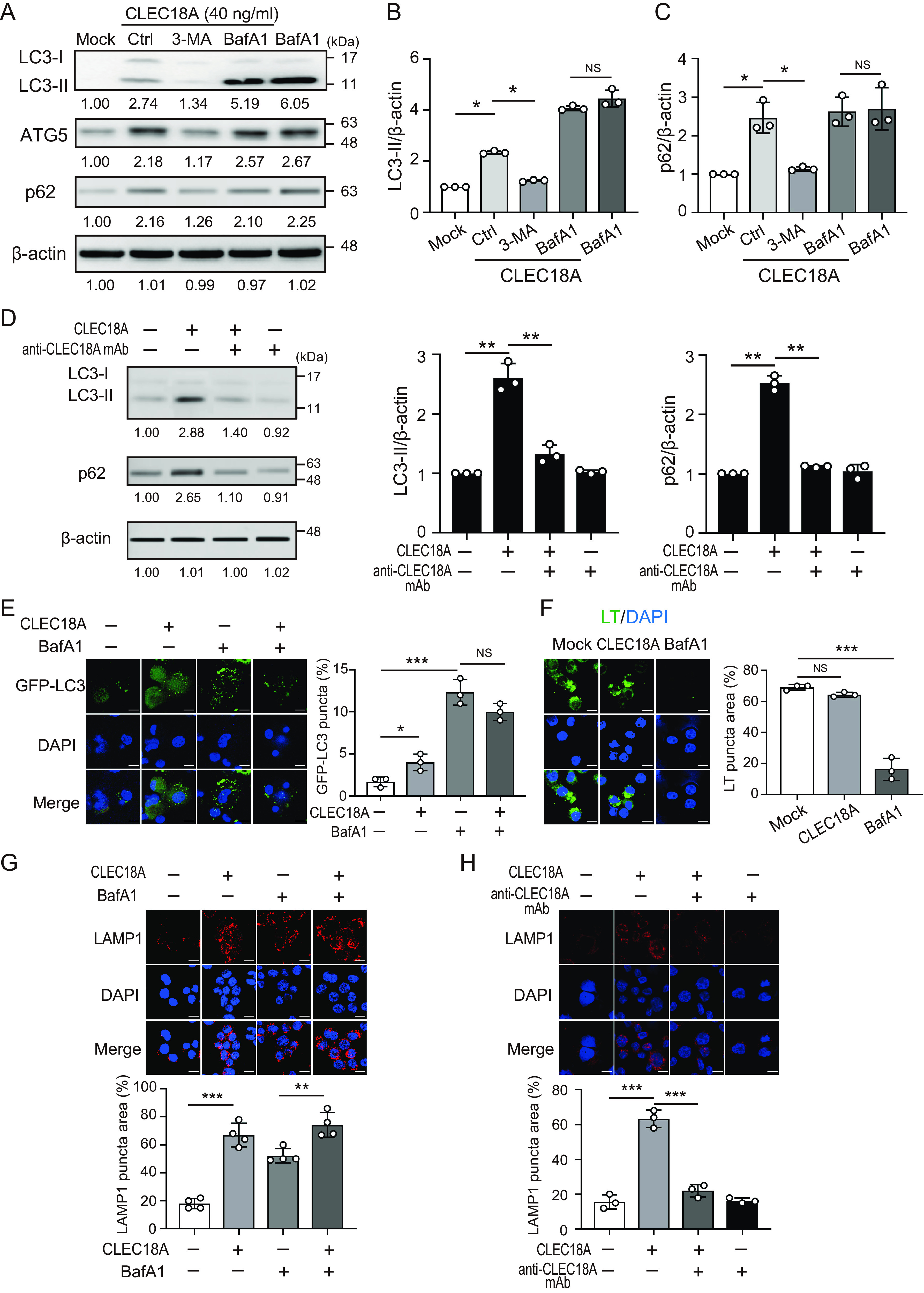
CLEC18A suppressed the fusion of autophagosomes with lysosomes. (A to C) THP-1-derived macrophages were treated with (A) CLEC18A (40 ng/mL) in the absence or presence of 3-methyladenine (3-MA, 10 μM) or BafA1 (100 nM) for 24 h. The expression of LC3, ATG5, p62, and β-actin was detected by using immunoblotting, and the (B) LC3-II/β-actin and (C) p62/β-actin ratios were calculated. (D) THP-1-derived macrophages were treated with CLEC18A (40 ng/mL) in the absence or presence of anti-CLEC18A antibodies (10 μg/mL) for 24 h. The expression of LC3, p62, and β-actin was detected and quantified by using immunoblotting. (E) THP-1 cells stably expressing the GFP-LC3 fusion protein were treated with the indicated reagent for 24 h. GFP-LC3 puncta were detected via confocal microscopy and quantified. (F) THP-1-derived macrophages were treated with CLEC18A (40 ng/mL) or BafA1 (100 nM) for 4 h and were stained with LysoTracker Green (LT, 2 μM). LT-positive puncta were detected via confocal microscopy and quantified. (G and H) THP-1-derived macrophages were treated with CLEC18A (40 ng/mL) in the absence or presence of (G) BafA1 (100 nM) for 4 h or (H) anti-CLEC18A antibodies (10 μg/mL) for 24 h and stained with an anti-LAMP1 antibody. LAMP1-positive puncta were detected via confocal microscopy and quantified. All of the experiments were performed in triplicate, and the data are presented as the mean ± SD. The scale bar in the IFA image represents 10 μm. *, *P *< 0.05; **, *P *< 0.01; ***, *P *< 0.005; NS, not significant.

Next, we explored how CLEC18A affects autophagic flux. As shown in Fig. 5A–C, BafA1 inhibited autophagosome degradation and caused LC3-II and p62 accumulation. However, neither LC3-II nor p62 increased significantly in THP-1 cells treated with CLEC18A in the presence of BafA1, suggesting that CLEC18A does not affect autophagosome formation in phagocytes. Moreover, CLEC18A-induced accumulation of LC3-II and p62 were both suppressed in the presence of anti-CLEC18A antibodies (10 μg/mL) (Fig. 5D). To further confirm our result, we analyzed autophagosome formation in THP-1 cells that were stably expressing the GFP-LC3 fusion protein (Fig. 5E). Although there were more GFP-LC3 puncta in the cells treated with BafA1, there was not a significant increase in GFP-LC3 puncta in the CLEC18A-treated cells after the addition of BafA1. This result raised the possibility of CLEC18A blocking autophagy after autophagosome formation, thereby leading to GFP-LC3 puncta accumulation.

BafA1 blocks autophagosome-lysosome fusion through inhibiting acidification. To examine whether CLEC18A inhibits autophagy by affecting acidification, we treated THP-1 cells with CLEC18A or BafA1 for 4 h and then incubated them with a specific fluorescent stain (LysoTracker Green) for the detection of acidic compartments within a cell. BafA1 decreased the number of acidic structures in the cell, thereby causing a rapid decrease in LysoTracker Green puncta (Fig. 5F). The acidification ratio did not significantly decrease in the cells treated with CLEC18A (64.3% ± 1.5%), compared with the control cells (69.0% ± 1.7%), indicating that CLEC18A does inhibit autophagy but that it does not do so by suppressing acidification.

Given that autophagy terminates with the degradation of autophagosome content in lysosomes, we analyzed the effect of CLEC18A on lysosomal degradation by detecting the subcellular distribution of the lysosomal-associated membrane protein 1 (LAMP1). There was an increase in the area of LAMP1-positive structures in cells treated with CLEC18A (67.0% ± 8.4%, *P *< 0.005) (Fig. 5G) or BafA1 (52.3% ± 5.2%, *P *< 0.01), compared with control cells (18.0% ± 3.6%). Moreover, BafA1-induced LAMP1 distribution significantly increased in cells cotreated with CLEC18A (74.3% ± 8.8% versus 52.3% ± 5.2%, *P *< 0.05). The CLEC18A induced LAMP1-positive structures were reduced in the presence of anti-CLEC18A antibodies (63.3% ± 5.0% versus 22.0% ± 3.6%, *P *< 0.005) (Fig. 5H). The result indicates that CLEC18A may block autophagy after autophagosome formation but before lysosomal degradation.

### CLEC18A has no significant impact on the Golgi complex.

Given that CLEC18A is located in endosomes (9), we analyzed the distribution of endo-lysosomal protein markers upon treatment with CLEC18A to examine whether these proteins could block the maturation of autophagosomes. The localization of cis-Golgi (GM130) and trans-Golgi (TGN46) marker proteins was unchanged after CLEC18A treatment, compared with control cells (Fig. S4), suggesting that CLEC18A has no significant impact on the Golgi complex in phagocytosis.

### CLEC18A inhibits autophagosome-lysosome fusion by decreasing the recruitment of Rab7 to autophagosomes.

We further explored the distribution of endosomal protein markers (e.g., EEA1, Rab5, and Rab7) upon treatment with either CLEC18A or starvation by using immunofluorescence microscopy. As shown in Fig. S5, there was no significant difference in the level of colocalization of EEA1 (Fig. S5A) or Rab5 (Fig. S5B) with LC3-positive puncta in the cells treated with CLEC18A, compared with those under starvation.

**FIG 6:**
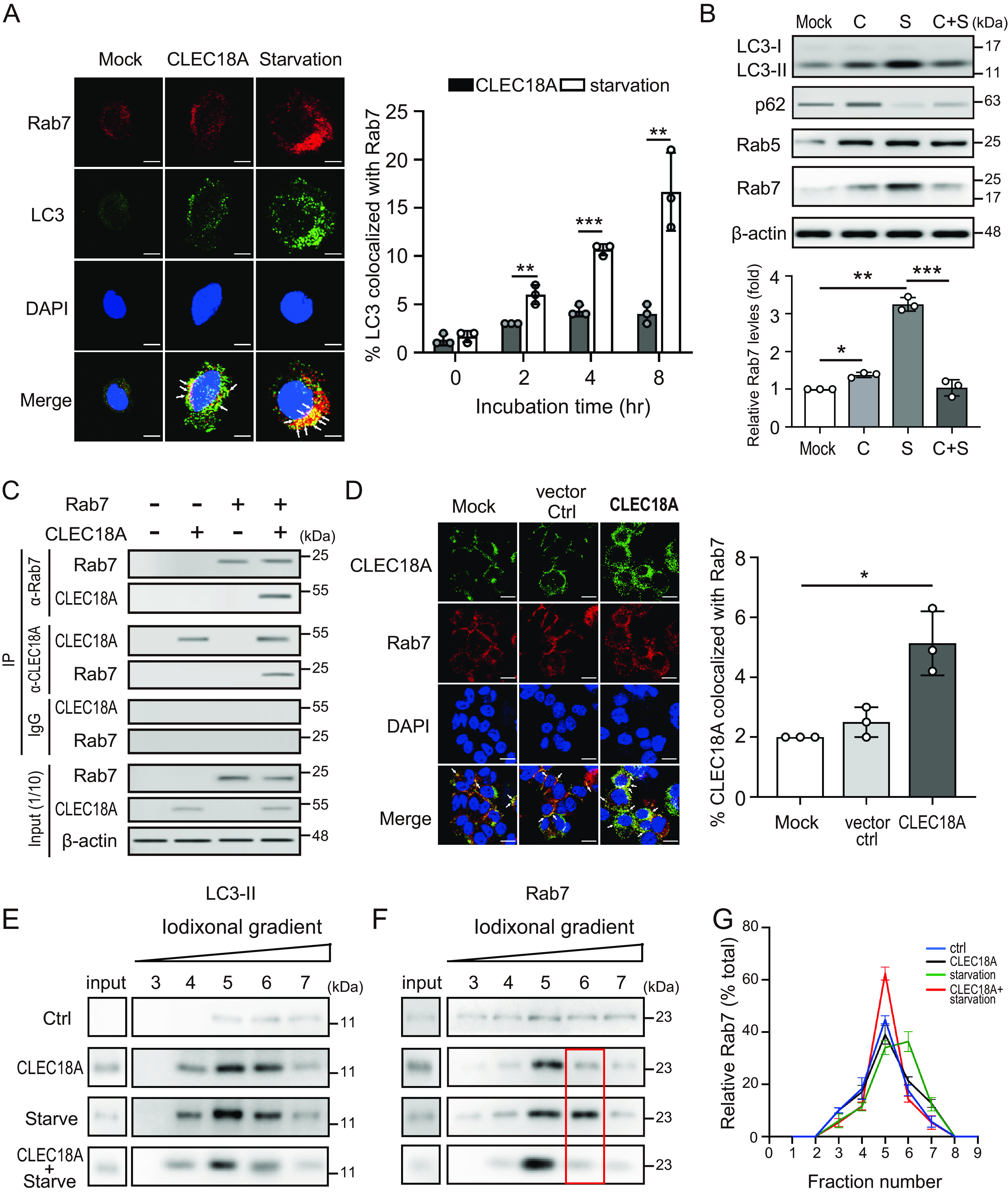
CLEC18A inhibits autophagosome-lysosome fusion by decreasing the recruitment of Rab7 to autophagosomes. (A) THP-1-derived macrophages were starved in amino acid-free medium or treated with CLEC18A (40 ng/mL) for the indicated times. The cells were stained with antibodies against LC3 (green) and Rab7 (red). The colocalization of Rab7 and LC3 puncta was detected via confocal microscopy (left panel, the represented image: 4 h) and calculated (right panel). (B) THP-1-derived macrophages were incubated in complete medium (mock), amino acid-free medium (starvation), complete medium with 40 ng/mL CLEC18A for 24 h, or complete medium with 40 ng/mL CLEC18A for 24 h with amino acid starvation for 24 h (CLEC18A + starvation). The expression of LC3, Rab5, and Rab7 was analyzed and quantified by using immunoblotting. C, CLEC18A; S, starvation. (C) Reciprocal immunoprecipitation assays revealed that CLEC18A specifically interacts with Rab7. 293T cells were mock transfected or transfected with pCMV-Myc-DDK tagged Rab7 and pCMV-CLEC18A. At 24 hours posttransfection, the cell lysates were subjected to immunoprecipitation (IP) with anti-Rab7, anti-CLEC18A antibodies, or rabbit IgG (negative control). Immunoprecipitated proteins were separated and then visualized via immunoblotting with an anti-Rab7 antibody or an anti-CLEC18A antibody. (D) THP-1-derived macrophages were mock transfected or transfected with pCMV-CLEC18A or vector control. At 24 hours posttransfection, the colocalization of CLEC18A and Rab7 was detected via confocal microscopy. (E to G) THP-1-derived macrophages were treated with the indicated reagent for 24 h. The cell homogenates were subjected to iodixanol density gradient centrifugation (10% to 50% gradient) and fractionated. The expression of (E) LC3 and (F) Rab7 was detected by using immunoblotting and (G) quantified. All the experiments were performed in triplicate, and the data are presented as the mean ± SD. The scale bar in the IFA image represents 10 μm. *, *P *< 0.05; **, *P *< 0.01; ***, *P *< 0.005.

Rab7 has been implicated in the fusion of autophagosomes with endosomes to form amphisomes before their fusion with lysosomes (17). We observed a significantly increased colocalization percentage of Rab7 with LC3-positive puncta (yellow puncta) in cells under starvation (8 h: 17.33% ± 3.22%, *P *< 0.01), compared with control cells (Fig. 6A). Notably, the colocalization seemed to be retarded in the cells treated with CLEC18A (8 h: 4.00% ± 1.00%, *P *< 0.05), despite the presence of numerous LC3 puncta (green puncta). We further examined the expression of Rab7 in cells treated with CLEC18A by using immunoblotting. There was only slightly increased Rab7 expression induced in cells upon CLEC18A treatment (1.38 ± 0.07-fold, *P *< 0.05) (Fig. 6B) compared with control cells. While Rab7 expression increased dramatically under starvation (3.25 ± 0.18-fold, *P *< 0.01), the induction was suppressed in the presence of CLEC18A (1.04 ± 0.21-fold, *P *< 0.005). Of note, CLEC18A had no effect on Rab5 expression, but it did increase p62 accumulation (2.29 ± 0.12-fold, *P *< 0.01) (Fig. 6B; Fig. S8). Moreover, the starvation-induced p62 degradation is significantly blocked by CLEC18A treatment (0.49 ± 0.13-fold versus 0.91 ± 0.02-fold, *P *< 0.05).

Next, we performed reciprocal immunoprecipitation experiments to validate the interactions between CLEC18A and Rab7. Cell lysates were prepared from 293T cells that were transiently expressing Myc-DDK-tagged Rab7 (OriGene, USA), and CLEC18A. The lysates were immunoprecipitated with anti-Rab7 and anti-CLEC18A antibodies or rabbit IgG (negative control), respectively. As shown in Fig. 6C, CLEC18A specifically coprecipitated with Rab7. Moreover, antibodies specific to CLEC18A also coprecipitated with Rab7, indicating that CLEC18A and Rab7 interact. The immunofluorescence assay revealed the increased colocalization of CLEC18A and Rab7 in CLEC18A-overexpressing cells, compared with control cells (Fig. 6D).

Based on our results, we hypothesized that overexpressed CLEC18A binds Rab7 and arrests Rab7 recruitment to autophagosomes. Therefore, we conducted a subcellular fractionation experiment to analyze the effect of CLEC18A on the density of membrane-bound LC3-II and Rab7 by using discontinuous iodixanol density gradient centrifugation. As shown in Fig. 6E, although both CLEC18A and starvation induced higher LC3-II expression, as expected, they had no effect on the density of membrane-bound LC3-II. Under normal conditions, the majority of Rab7 was in fraction 5, as shown in the control cells (Fig. 6F and G). Upon starvation, it shifted to the denser fraction 6. While CLEC18A treatment had no effect on the Rab7 density distribution, it blocked the starvation-induced shift of Rab7 to fraction 6, suggesting that it may decrease the recruitment of Rab7 to autophagosomes.

Rab7 is present on late endosomes, and the gain of Rab7 on late endosomes is accompanied by the loss of Rab5, which is known as the Rab5-to-Rab7 switch (18). We further examined whether CLEC18A interacts with Rab5. CLEC18A did interact with Rab5 (Fig. S6A and B), but it had no effect on the Rab5 density distribution (Fig. S6C).

### CLEC18A does not inhibit endocytosis.

In addition to autophagy, Rab7 is involved in the regulation of endocytosis (17). Both autophagy and endocytosis are associated with the absorption of extracellular material, and they have converging steps and common participating molecules (19). We explored the effect of CLEC18A on endocytosis by measuring the fluorescent dextran uptake in cells treated with CLEC18A. As shown in Fig. S7A, there was no significant difference in the level of dextran in the lysosomes of the CLEC18A-treated cells, compared with the control cells. We also examined whether CLEC18A affects endocytic trafficking and membrane fusion or lysosomal hydrolytic function by measuring its effects on epidermal growth factor receptor (EGFR) turnover. There was no significant difference in endocytosis-mediated EGFR degradation in the cells treated with CLEC18A, compared with the control cells (Fig. S7B).

## DISCUSSION

**FIG 7:**
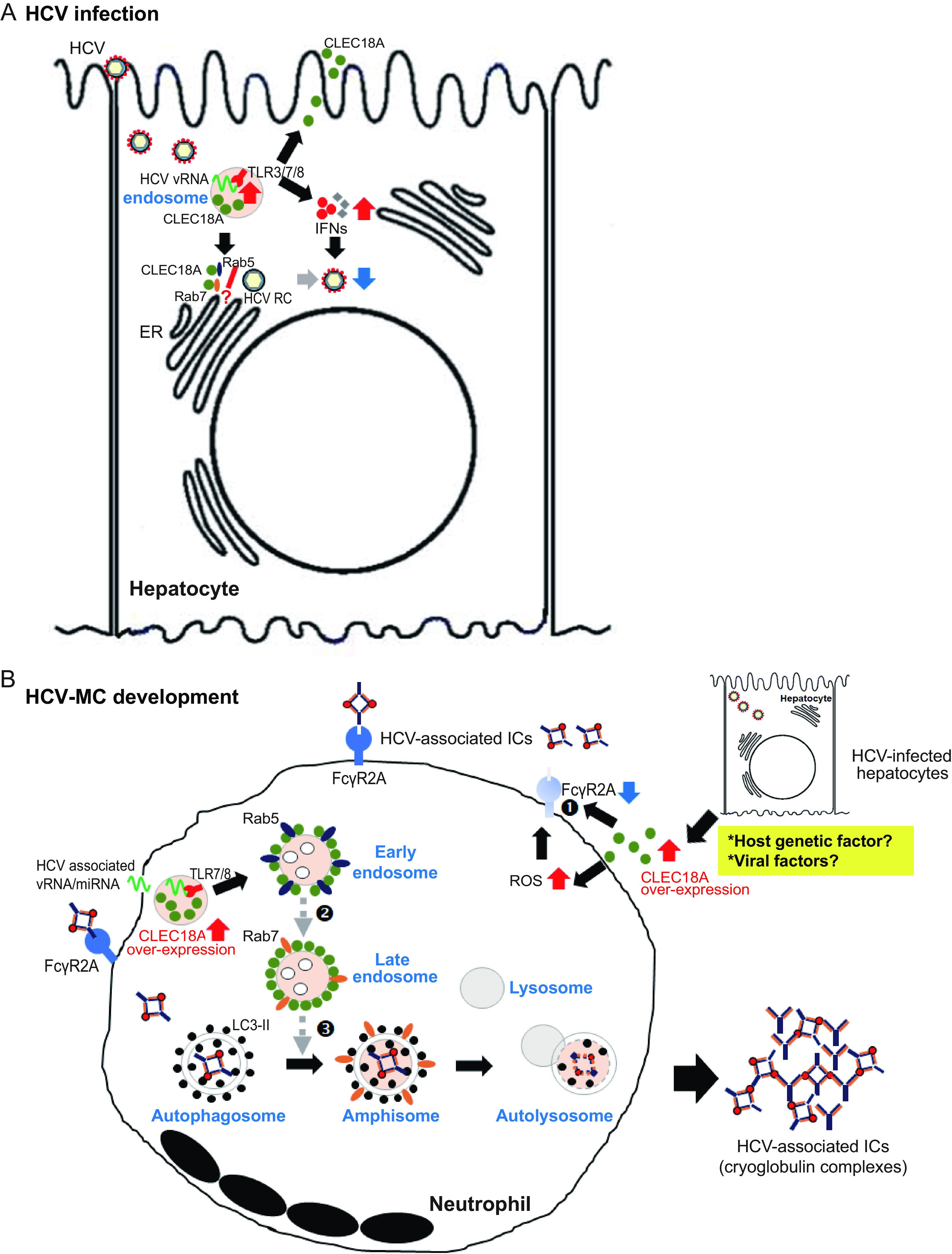
Proposed model for the biological role of CLEC18A in (A) HCV infection and (B) HCV-associated MC development, based on the results of this study. (A) In early infection, HCV induces CLEC18A expression in hepatocytes through TLR3/7/8 activation. CLEC18A interacts with Rab5 and Rab7, perhaps interfering with their binding to the HCV replication complex (RC), thereby inhibiting HCV replication. In addition, upregulated CLEC18A enhances the expression of type I/III interferons (IFNs) upon TLR3 activation to protect against HCV infection. (B) Host genetics or viral factors may induce CLEC18A overexpression. Excessively upregulated CLEC18A impairs the uptake and clearance of ICs and causes HCV-associated MC development by (i) inhibiting FcγRIIA expression; (ii) interacting with Rab5 to interfere in the small GTPase exchange activity, thereby suppressing Rab7 expression; and (iii) reducing the recruitment of Rab7 to autophagosomes, thereby retarding autophagosome maturation and arresting autophagosome-lysosome fusion, thereby resulting in immune complex accumulation.

Autophagy is a natural defense mechanism that clears microbial infections and thus plays a key role in dominating invading pathogens (20). However, many pathogens have also developed strategies against this intracellular antimicrobial mechanism and even use this pathway to enhance their own replication. Accumulating evidence demonstrates that HCV enhances its replication by inducing autophagy (21, 22). Therefore, autophagy inhibitors have emerged as therapeutic candidates with which to suppress HCV infections. In this study, we identified that a novel cellular factor, namely, CLEC18A, is involved in HCV infection through an interaction with Rab5/7 and the enhancement of type I/III IFN expression to inhibit viral replication (Fig. 7A). We observed increased CLEC18A levels in Huh7.5 cells after HCV infection. The downregulation of cellular CLEC18A enhanced HCV replication. Conversely, CLEC18A overexpression inhibited viral replication, suggesting that CLEC18A can inhibit virus production. Previous studies have demonstrated that Rab proteins interact with the HCV replication complex and are crucial for HCV replication (23, 24). Su et al. (25) demonstrated that Rab5 and class III phosphoinositide 3-kinases (PI3K) vacuolar sorting protein 34 (Vps34) formed a complex with HCV NS4B. Downregulated Rab5 resulted in a significant reduction in NS4B-induced or HCV-induced autophagic vesicle formation (25). We found that CLEC18A interacts with Rab5 and Rab7. We presume that this interaction may interfere in the binding of Rab5/7 and the HCV replication complex to thereby suppress HCV replication. Further in-depth studies are needed to confirm our hypothesis. On the other hand, type I and III IFNs are key cellular factors to protect against HCV infection (26). We recently identified that CLEC18A is a TLR3 coreceptor and enhances the host immune response during influenza virus infection (10). In the present study, we showed that CLEC18A could enhance type I and type III IFN production in hepatocytes upon TLR3 activation to inhibit HCV replication. More in-depth experiments are required to dissect how CLEC18A regulates the expression of IFNs during HCV replication.

During infection, the host immune system is induced to produce several cellular factors to protect against pathogen invasion. However, when the immune response overreacts and there is dysregulated cytokine homeostasis, autoimmunity occurs, following the infection. Approximately 50% of patients with HCV exhibit a wide range of MC symptoms (27). The reasons why MC appears in only a portion of patients with HCV are still unclear, although host genetics or viral factors have been postulated as key determinants (27, 28). We previously demonstrated increased CLEC18A expression in the phagocytes and sera of patients with HCV infections, particularly those with MC symptoms (12). Herein, we demonstrated that TLR 3/7/8 activation induced CLEC18A upregulation. Mosaad et al. (29) demonstrated that the TLR3 genotype may be a susceptibility risk factor for chronic HCV infection. Several reports have demonstrated that TLR7 polymorphism is associated with chronic HCV infections as well as with the outcomes of patients (30–32). We recently reported that patients with HCV-associated MC who carried G-alleles of the TLR7 single nucleotide polymorphism (SNP) rs3853839 had higher TLR7 and B-cell activating factor (BAFF) expression (28). Newman et al. (33) demonstrated that the codon optimization of TLR7 increases protein levels as well as the response to ligands. In the present study, we demonstrated that stimulation with a TLR7/8 ligand induced CLEC18A expression in neutrophils. The association between TLR7 polymorphism and CLEC18A expression needs to be confirmed with additional large-scale or in-depth studies. In addition to host genetic factors, the HCV genotype may be another factor that affects the level of CLEC18A induction. The association between the genotype of human TLRs/HCV and the level of CLEC18A induction requires additional large-scale investigation.

MC is characterized by the abnormal presence of large ICs (cryoglobulins) that may be due to the reduced uptake and clearance of ICs. Fc receptors (FcRs) are important in both promoting and regulating the immune and inflammatory responses to ICs (7, 8). Moreover, FcRs play a crucial role in the clearance of ICs, which leads to the degradation of antigen-antibody complexes, as well as directs the antigenic peptides to the major histocompatibility complex (MHC) class I or class II antigen presentation pathway (34). FcRs differ in their antibody affinities, depending on their molecular structures, which allows for the activation of FcγR type I (FcγRI) by a monomeric IgG (high affinity), whereas FcγRII and FcγRIII are required to bind multiple IgG molecules within an IC to be activated (low affinity) (8, 35). Previous studies have indicated that the clearance of circulating cryoglobulins should be mediated by low-affinity FcγRs (e.g., FcγRII and FcγRIII), which are mainly on phagocytes (8). Both nucleotide polymorphisms and gene copy number variants of FcγRs can affect their interactions with antibodies (34). However, a previous report revealed no significant difference in FcγR genotypes in patients with MC (36).

In addition to MC, circulating immune complexes (ICs) play a key role in SLE pathogenesis through the activation of FcγR, complement, and intracellular TLRs (37, 38). Sturfelt et al. (39) proposed that the impaired complement function in SLE patients causes accelerating cell apoptosis, the release of nuclear antigens, the formation of autoantibodies, and the eventual accumulation of ICs. Lood et al. (16) demonstrated that TLR7/8 activation in neutrophils from SLE patients impairs ICs phagocytosis through the shedding of FcγRIIA. We demonstrated the increased expression of CLEC18A in human neutrophils after stimulation with the TLR7/8 ligand R848, which was accompanied by decreased FcγRIIA expression. When the intracellular CLEC18A was knocked down, the R848-induced decrease of FcγRIIA was rescued. Our results suggest that CLEC18A may be involved in TLR7/8 activation-associated FcγRIIA shedding. However, the role of CLEC18A in SLE pathogenesis requires further experiments for dissection.

In this study, we found that FcγRIIA was significantly decreased in neutrophils from patients with HCV-associated MC, compared with those without MC or healthy controls. We found that upregulated CLEC18A could inhibit FcγRIIA expression to impair phagocytosis in a dose-dependent manner, which is associated with NOX2-dependent ROS production (Fig. 7B). This suggests that overexpressing CLEC18A-induced ROS may be associated with the development of MC. The detailed mechanism by which CLEC18A regulates FcγRIIA expression needs to be elucidated with additional experimentation. On the other hand, the binding of Fc receptors to immunoglobulins is highly dependent upon the glycosylation of the antibodies. CLEC18A has low affinity to monosaccharides, and we have not found that it can bind to human IgG on an ELISA binding assay (9). Moreover, our previous result showed that there was no significant difference in CLEC18A levels in circulating B cells between MC patients and healthy control subjects (12). These observations suggest that the CLEC18A-mediated inhibition of phagocytosis is not via binding to immunoglobulins.

The degradation of macromolecules (e.g., cryoglobulins) in cells could enter the endosomal-lysosomal system via autophagy, endocytosis, and/or phagocytosis (40). Our *in vitro* cell-based results demonstrated that overexpressed CLEC18A has no effect on endocytosis and does not affect autophagosome formation but does suppress phagocytosis. We showed that overexpressed CLEC18A could interact with Rab5 and Rab7, respectively. Rab7 is a member of the Rab family of small GTPases and is predominantly located on late endosomes, autophagosomes, and lysosomes (41). It is a key factor in the organization of effector proteins into specific membrane subdomains (42), and it is involved in the transport of endosomes from early to late endocytic compartments of the cell to contribute to effective autophagy and endocytosis (42, 43). All Rabs alternate between an active (GTP-bound) state and an inactive (GDP-bound) state (44). This molecular switch is strictly regulated. Poteryaev et al. (45) identified Mon1/SAND-1 as an important regulator in the Rab5-to-Rab7 conversion process, and it is also actively involved in the recruitment of Rab7 to endosomes. Our results revealed that CLEC18A has no effect on Rab5 expression and does not affect its recruitment to autophagosomes. However, CLEC18A suppressed starvation-induced Rab7 expression and inhibited the recruitment of Rab7 to autophagosomes, suggesting that CLEC18A may affect Rab7 expression/activation and retard phagosome maturation. We hypothesize that the overacted interaction between CLEC18A and Rab5 may interfere in the small GTPase exchange activity, which affects the expression and activation of Rab7 by modulating the Rab5-to-Rab7 transition. In addition, the interaction between CLEC18A and Rab7 may affect the binding efficiency of Rab7 to autophagosomes, resulting in the slowdown of phagosome maturation. Retarding autophagosome maturation could affect autophagosome-lysosome fusion, resulting in reduced phagocytotic activity for the clearance of ICs (Fig. 7B). Further in-depth studies are needed to confirm our hypothesis.

To the best of our knowledge, this report is the first pilot study to evaluate the biological function of CLEC18A in HCV infection and HCV-EHMs. Although we have revealed several novel findings, this study has some limitations. First, the study included a small number of cases. Therefore, it is not likely to reflect the complete characteristics of chronic HCV infections. Second, this study was cross-sectional in design. Thus, we cannot rule out the possibility that the CLEC18A expression changed as a result of the therapeutic strategies. Finally, although we performed *in vitro* cell-based assays to demonstrate increased CLEC18A expression in HCV-infected cells, compared with uninfected cells, the positive control was lacking. Future studies focusing on CLEC18A *ex vivo* and an in-depth analysis of the pathogenic mechanisms in HCV infections are needed.

We observed a decreased trend in the HCV RNA titer and cryoglobulin levels in the sera of HCV-MC patients after DAAs therapy (Fig. S1A and B). Additionally, the dynamic change in CLEC18A levels was positively correlated with the change in HCV RNA levels (Fig. S1C), which is consistent with our previously published results (12). Based on our results, we propose that CLEC18A may be used as a marker to evaluate the effect of anti-HCV therapeutic drugs and could be a potential predisposing factor for the development of MC syndrome. Further large cohort studies are required to confirm our observation. Herein, we offer new molecular machinery with which to understand the association between HCV infection and autoimmunity.

## MATERIALS AND METHODS

### Subjects.

This prospective study was conducted at a medical center from 2016 to 2021. A total of 120 participants, including 42 patients with HCV-associated MC, 18 patients with HCV infection but without MC (non-MC), 26 rheumatic patients without infection, and 34 healthy subjects, were enrolled from the Taichung Veterans General Hospital in Taiwan. The enrolled patients fulfilled the 2002 revised version of the European criteria for Sjögren syndrome (SS) (46), the 2010 revised criteria of the American College of Rheumatology (ACR) for rheumatoid arthritis (RA) (47), and the 1997 revised criteria of the ACR for systemic lupus erythematosus (SLE) (48). Among the 26 rheumatic patients, 14 had SS, 10 had RA, and 2 had SLE. The serum alanine aminotransferase (ALT), serologic test for HCV (anti-HCV antibodies), and HCV viral load were measured. The Institutional Review Board of Taichung Veterans General Hospital approved this study (SF16036B), and the written consent of all of the participants was obtained according to the Declaration of Helsinki.

### Determination of serum cryoglobulinemia.

The diagnosis of cryoglobulinemia was defined by the presence of cryoglobulins in the serum stored at 4°C for 10 days in two fractions as well as the reversibility of the cryoprecipitation in one fraction replaced at 37°C when a cryoprecipitate was formed (27). The classification of serum cryoglobulinemia was determined via immuno-electrophoresis (27).

### Cell culture.

The peripheral blood mononuclear cells (PBMCs) were immediately isolated from venous blood using Ficoll-Paque PLUS (GE Healthcare Biosciences AB, Uppsala, Sweden) density gradient centrifugation. To isolate the neutrophils, heparinized blood was layered on a Polymorphprep (Axis-Shield, Dundee, United Kingdom) density gradient, according to the manufacturer’s instructions. The red blood cells were lysed with a hypertonic solution, while the PBMCs, neutrophils, and THP-1 or HL60 (ATCC CCL-240) cells were suspended in RPMI 1640 medium (Thermo Fisher Scientific, Fremont, CA, USA) supplemented with 10% FBS, 1× nonessential amino acids, 100 units/mL penicillin, 100 units/mL streptomycin, and 2% autologous serum (for PBMCs or PMNs culture only) in an incubator containing 5% CO_2_ at 37°C. To readily induce differentiation into macrophages, the THP-1 cells were grown in media and treated with 10 ng/mL phorbol myristate acetate (PMA) (Sigma-Aldrich, St. Louis, MO, USA) overnight. To readily induce differentiation into neutrophil-like cells (dHL-60), the HL-60 cells were grown in media and treated with 1.3% DMSO (Sigma-Aldrich) for 72 h.

### Phagocytosis assay.

A phagocytosis assay was carried out using a Phagocytosis Assay Kit (Cayman, USA), according to the manufacturer’s instructions. The assay employed latex beads coated with fluorescently labeled rabbit IgG as a probe to measure the phagocytic process *in vitro*. The cells were incubated with the latex beads-rabbit IgG-FITC complex at 37°C for 1 h. The engulfed fluorescent beads were detected using a fluorescence microscope and flow cytometry. Phagocytosis was quantified via flow cytometry.

### Flow cytometry analysis.

The intracellular staining of CLEC18A was performed, following fixation and permeabilization with the IntraPrep Permeabilization Reagent (Beckman Coulter, Brea, CA, USA), using the modified method of a previous study (12). Cells were incubated with the Alexa Fluor 647-conjugated anti-CLEC18A monoclonal antibody (clone 3A9E6), and Alexa Fluor 647-conjugated IgG1 (R&D Systems, Minneapolis, USA) was used as an isotype control. For the analysis of the cell surface expression of FcγRIIA, human neutrophils were treated with or without recombinant CLEC18A protein (40 ng/mL, MyBioSource, San Diego, USA) for 4 h. This was followed by FITC-conjugated anti-FcγRIIA monoclonal antibody (clone IV.3, Stem Cell Technologies, Columbia, Canada) incubation, and FITC-conjugated IgG2b (Stem Cell Technologies) was used as an isotype control. The cells were examined via flow cytometry (FACSCanto II, BD Biosciences, San Jose, CA, USA). The data were analyzed using the CellQuest software package and were expressed as the mean fluorescence intensity (MFI).

### Quantitative reverse transcription-PCR.

The total RNAs were extracted using the TRIzol Reagent (Thermo Fisher Scientific) and were purified using the RNeasy MinElute Cleanup Kit (Qiagen, Valencia, CA, USA), according to the manufacturer’s instructions. The purified RNAs were quantified at OD_260_ and 280 nm using a NanoDrop spectrophotometer (Thermo Fisher Scientific). For the mRNA detection, the total RNA was subjected to reverse transcription with an oligonucleotide (dT)_20_ primer to target the mRNA using the SuperScript first-strand synthesis system (Thermo Fisher Scientific), according to the manufacturer’s instructions. The single-stranded cDNA was subjected to qRT-PCR using a TaqMan Gene Expression Assay Kit (Thermo Fisher Scientific) with specific primer and probe sets. The glyceraldehyde 3-phosphate dehydrogenase (GAPDH) gene was used as an internal control. The qRT-PCRs were performed on a StepOnePlus real-time PCR system (Thermo Fisher Scientific), using standard protocol.

### Immunoblotting.

The cells with different treatments were lysed in a radio-immunoprecipitation assay (RIPA) buffer (25 mM Tris-HCl [pH 7.6], 150 mM NaCl, 1% NP-40, 1% sodium deoxycholate, and 0.1% SDS) that contained a protease inhibitor cocktail (Complete, Roche, Germany). 20 μg of total protein from an exosome lysate were loaded and separated on a standard sodium dodecyl sulfate (SDS)-polyacrylamide gel electrophoresis (PAGE) gel and were transferred to a polyvinylidene difluoride (PVDF) membrane (Millipore, USA). The membranes were incubated with primary antibodies, and this was followed by peroxidase-conjugated secondary antibodies. The results were detected using a charge-coupled device (CCD) camera-based imager (GE Healthcare Life Sciences) after membrane incubation with enhanced chemiluminescence (ECL) substrates (Millipore).

### Immunofluorescence assay.

THP-1 cell-derived macrophages with individual treatments were fixed with 4% paraformaldehyde at room temperature for 10 min, and they were then washed three times with phosphate-buffered saline (PBS). The cells were permeabilized in PBS containing 1% BSA and 0.2% saponin, and they were then blocked for 1 h in PBS containing 2% BSA. They were then incubated with the primary antibodies, and this was followed by the secondary antibodies. Coverslips were mounted onto glass slides with DAPI that contained the SlowFade mounting medium (Thermo Fisher Scientific), and the images were observed and recorded on an Olympus FV1000 laser scanning confocal microscope. The images were analyzed using the FV10-ASW version 4.2 software package. For the quantification of cells showing LC3-positive vesicles, approximately 50 cells were counted, and the cells with more than 20 LC3-labeled puncta were labeled as having formed autophagosomes.

### ROS analysis.

The human neutrophils or dHL60 cells were incubated with the NOX2 inhibitor diphenyleneiodonium (DIP) (25 μM) for 30 min before the addition of CLEC18A (40 ng/mL) for an additional 60 min. The fluorescent dye dihydrorhodamine (DHR) 123 (30 μM; Thermo Fisher Scientific) was added during the last 30 min of incubation, and the reactive oxygen species (ROS) were analyzed via flow cytometry (FACSCanto II, BD Biosciences). The flow cytometry data were analyzed using the CellQuest software and were expressed as the mean fluorescence intensity (MFI) of the cytosolic ROS.

### Density gradient centrifugation.

The gradient centrifugation was performed by using the modified method of a previous study (49). THP-1 cell-derived macrophages were treated with complete media in the absence or presence of CLEC18A (40 ng/mL) for 24 h. The starvation-treated cells were washed and incubated in amino acid-free media at the final hour. The cells were placed on ice, washed with PBS, and collected in HB buffer (25 mM Tris [pH 7.5], 250 mM sucrose, 50 mM NaCl, 1 mM EDTA) containing protease inhibitors. The samples were homogenized by passing through a 25-gauge needle and were centrifuged at 1,000 × *g* for 5 min. The supernatant was collected and loaded on the top of a discontinuous iodixonal (Optiprep, Stem Cell Technologies) gradient that ranged from 10% to 50%. The samples were centrifuged at 100,000 × *g* at 4°C for 8 h. Following centrifugation, the fractions were collected and subjected to SDS-PAGE and immunoblotting.

### Epidermal growth factor receptor degradation assay.

THP-1 cell-derived macrophages were incubated in serum-free RPMI for 3 h. Then, 40 ng/mL CLEC18A (or PBS control) were added for an additional 2 h. The medium was then replaced with serum-free RPMI containing 40 ng/mL EGF and 20 mg/mL cycloheximide, with or without 40 ng/mL CLEC18A or 100 nM BafA1, for the indicated times. The cells were lysed and subjected to an immunoblotting analysis.

### Statistical analysis.

An unpaired, two-tailed Student’s *t* test was used for the between-group comparisons. A one-way analysis of variance (ANOVA) with the *post hoc* Bonferroni test or Dunnett’s test was used for the multiple comparisons. The correlation coefficient was calculated using Spearman’s correlation test. *P* values of <0.05 were regarded as indicative of a statistically significant result, and the tests were performed using GraphPad Prism 8.

## References

[1] Lauer GM, Walker BD. 2001. Hepatitis C virus infection. N Engl J Med 345:41–52. doi:10.1056/NEJM200107053450107. 11439948

[2] Cacoub P, Poynard T, Ghillani P, Charlotte F, Olivi M, Piette JC, Opolon P, for the MULTIVIRC GROUP. 1999. Extrahepatic manifestations of chronic hepatitis C. MULTIVIRC Group Multidepartment Virus C Arthritis Rheum 42:2204–2212. doi:10.1002/1529-0131(199910)42:10<2204::AID-ANR24>3.0.CO;2-D. 10524695

[3] Cacoub P, Gragnani L, Comarmond C, Zignego AL. 2014. Extrahepatic manifestations of chronic hepatitis C virus infection. Dig Liver Dis 46:S165–S173. doi:10.1016/j.dld.2014.10.005. 25458776

[4] Ramos-Casals M, Muñoz S, Medina F, Jara LJ, Rosas J, Calvo-Alen J, Brito-Zerón P, Forns X, Sánchez-Tapias JM, HISPAMEC Study Group. 2009. Systemic autoimmune diseases in patients with hepatitis C virus infection: characterization of 1020 cases (The HISPAMEC Registry). J Rheumatol 36:1442–1448. doi:10.3899/jrheum.080874. 19369460

[5] Charles ED, Dustin LB. 2009. Hepatitis C virus-induced cryoglobulinemia. Kidney Int 76:818–824. doi:10.1038/ki.2009.247. 19606079 PMC2755598

[6] Ramos-Casals M, Stone JH, Cid MC, Bosch X. 2012. The cryoglobulinaemias. Lancet 379:348–360. doi:10.1016/S0140-6736(11)60242-0. 21868085

[7] Nimmerjahn F, Ravetch JV. 2008. Fcgamma receptors as regulators of immune responses. Nat Rev Immunol 8:34–47. doi:10.1038/nri2206. 18064051

[8] Takai T. 2002. Roles of Fc receptors in autoimmunity. Nat Rev Immunol 2:580–592. doi:10.1038/nri856. 12154377

[9] Huang Y-L, Pai F-S, Tsou Y-T, Mon H-C, Hsu T-L, Wu C-Y, Chou T-Y, Yang W-B, Chen C-H, Wong C-H, Hsieh S-L. 2015. Human CLEC18 gene cluster contains C-type lectins with differential glycan-binding specificity. J Biol Chem 290:21252–21263. doi:10.1074/jbc.M115.649814. 26170455 PMC4571857

[10] Huang YL, Huang MT, Sung PS, Chou TY, Yang RB, Yang AS, Yu CM, Hsu YW, Chang WC, Hsieh SL. 2021. Endosomal TLR3 co-receptor CLEC18A enhances host immune response to viral infection. Commun Biol 4:229. doi:10.1038/s42003-021-01745-7. 33603190 PMC7893028

[11] Tsai TY, Peng CY, Yang HI, Huang YL, Tao MH, Yuan SS, Lai HC, Hsieh SL. 2018. The human C-type lectin 18 is a potential biomarker in patients with chronic hepatitis B virus infection. J Biomed Sci 25:59. doi:10.1186/s12929-018-0460-2. 30055605 PMC6064175

[12] Liao TL, Huang YL, Chen YM, Lee HC, Chen DY, Hsieh SL. 2018. Association of C-type lectin 18 levels with extrahepatic manifestations in chronic HCV infection. Sci Rep 8:17287. doi:10.1038/s41598-018-35774-w. 30470801 PMC6251874

[13] Huang JY, Su WC, Jeng KS, Chang TH, Lai MM. 2012. Attenuation of 40S ribosomal subunit abundance differentially affects host and HCV translation and suppresses HCV replication. PLoS Pathog 8:e1002766. doi:10.1371/journal.ppat.1002766. 22792060 PMC3394201

[14] Cacoub P, Comarmond C, Vieira M, Régnier P, Saadoun D. 2022. HCV-related lymphoproliferative disorders in the direct-acting antiviral era: from mixed cryoglobulinaemia to B-cell lymphoma. J Hepatol 76:174–185. doi:10.1016/j.jhep.2021.09.023. 34600000

[15] Hayashi F, Means TK, Luster AD. 2003. Toll-like receptors stimulate human neutrophil function. Blood 102:2660–2669. doi:10.1182/blood-2003-04-1078. 12829592

[16] Lood C, Arve S, Ledbetter J, Elkon KB. 2017. TLR7/8 activation in neutrophils impairs immune complex phagocytosis through shedding of FcgRIIA. J Exp Med 214:2103–2119. doi:10.1084/jem.20161512. 28606989 PMC5502427

[17] Hyttinen JMT, Niittykoski M, Salminen A, Kaarniranta K. 2013. Maturation of autophagosomes and endosomes: a key role for Rab7. Biochim Biophys Acta 1833:503–510. doi:10.1016/j.bbamcr.2012.11.018. 23220125

[18] Rink J, Ghigo E, Kalaidzidis Y, Zerial M. 2005. Rab conversion as a mechanism of progression from early to late endosomes. Cell 122:735–749. doi:10.1016/j.cell.2005.06.043. 16143105

[19] Tooze SA, Abada A, Elazar Z. 2014. Endocytosis and autophagy: exploitation or cooperation? Cold Spring Harb Perspect Biol 6:a018358. doi:10.1101/cshperspect.a018358. 24789822 PMC3996471

[20] Gutierrez MG, Master SS, Singh SB, Taylor GA, Colombo MI, Deretic V. 2004. Autophagy is a defense mechanism inhibiting BCG and Mycobacterium tuberculosis survival in infected macrophages. Cell 119:753–766. doi:10.1016/j.cell.2004.11.038. 15607973

[21] Tanida I, Fukasawa M, Ueno T, Kominami E, Wakita T, Hanada K. 2009. Knockdown of autophagy-related gene decreases the production of infectious hepatitis C virus particles. Autophagy 5:937–945. doi:10.4161/auto.5.7.9243. 19625776

[22] Chan ST, Ou JJ. 2017. Hepatitis C virus-induced autophagy and host innate immune response. Viruses 9:224. doi:10.3390/v9080224. 28805674 PMC5580481

[23] Stone M, Jia S, Heo WD, Meyer T, Konan KV. 2007. Participation of Rab5, an early endosome protein, in hepatitis C virus RNA replication machinery. J Virol 81:4551–4563. doi:10.1128/JVI.01366-06. 17301141 PMC1900164

[24] Manna D, Aligo J, Xu C, Park WS, Koc H, Heo WD, Konan KV. 2010. Endocytic Rab proteins are required for hepatitis C virus replication complex formation. Virology 398:21–37. doi:10.1016/j.virol.2009.11.034. 20005553 PMC2823978

[25] Su WC, Chao TC, Huang YL, Weng SC, Jeng KS, Lai MMC. 2011. Rab5 and class III phosphoinositide 3-kinase Vps34 are involved in hepatitis C virus NS4B-induced autophagy. J Virol 85:10561–10571. doi:10.1128/JVI.00173-11. 21835792 PMC3187495

[26] Bolen CR, Ding S, Robek MD, Kleinstein SH. 2014. Dynamic expression profiling of type I and type III interferon-stimulated hepatocytes reveals a stable hierarchy of gene expression. Hepatology 59:1262–1272. doi:10.1002/hep.26657. 23929627 PMC3938553

[27] Damoiseaux J. 2014. The diagnosis and classification of the cryoglobulinemic syndrome. Autoimmun Rev 13:359–362. doi:10.1016/j.autrev.2014.01.027. 24424176

[28] Liao TL, Chen YM, Hsieh SL, Tang KT, Chen DY, Yang YY, Liu HJ, Yang SS. 2021. Hepatitis C virus-induced exosomal microRNAs and TLR7 polymorphism regulate B-cell activating factor. mBio 12:e02764-21. doi:10.1128/mBio.02764-21. 34724826 PMC8561394

[29] Mosaad YM, Metwally SS, Farag RE, Lotfy ZF, AbdelTwab HE. 2019. Association between Toll-like receptor 3 (TLR3) rs3775290, TLR7 rs179008, TLR9 rs352140 and chronic HCV. Immunol Invest 48:321–332. doi:10.1080/08820139.2018.1527851. 30321082

[30] Schott E, Witt H, Neumann K, Taube S, Oh D-Y, Schreier E, Vierich S, Puhl G, Bergk A, Halangk J, Weich V, Wiedenmann B, Berg T. 2007. A Toll-like receptor 7 single nucleotide polymorphism protects from advanced inflammation and fibrosis in male patients with chronic HCV-infection. J Hepatol 47:203–211. doi:10.1016/j.jhep.2007.03.021. 17512627

[31] Schott E, Witt H, Neumann K, Bergk A, Halangk J, Weich V, Müller T, Puhl G, Wiedenmann B, Berg T. 2008. Association of TLR7 single nucleotide polymorphisms with chronic HCV-infection and response to interferon-a-based therapy. J Viral Hepat 15:71–78. doi:10.1111/j.1365-2893.2007.00898.x. 18088248

[32] Wang CH, Eng HL, Lin KH, Chang CH, Hsieh CA, Lin YL, Lin TM. 2011. TLR7 and TLR8 gene variations and susceptibility to hepatitis C virus infection. PLoS One 6:e26235. doi:10.1371/journal.pone.0026235. 22022576 PMC3192790

[33] Newman ZR, Young JM, Ingolia NT, Barton GM. 2016. Differences in codon bias and GC content contribute to the balanced expression of TLR7 and TLR9. Proc Natl Acad Sci USA 113:E1362–137. 26903634 10.1073/pnas.1518976113 PMC4791032

[34] Lu LL, Suscovich TJ, Fortune SM, Alter G. 2018. Beyond binding: antibody effector functions in infectious diseases. Nat Rev Immunol 18:46–61. doi:10.1038/nri.2017.106. 29063907 PMC6369690

[35] Smith KG, Clatworthy MR. 2010. Fc gamma RIIB in autoimmunity and infection: evolutionary and therapeutic implications. Nat Rev Immunol 10:328–343. doi:10.1038/nri2762. 20414206 PMC4148599

[36] Gragnani L, Piluso A, Giannini C, Caini P, Fognani E, Monti M, Petrarca A, Ranieri J, Razzolini G, Froio V, Laffi G, Zignego AL. 2011. Genetic determinants in hepatitis C virus-associated mixed cryoglobulinemia: role of polymorphic variants of BAFF promoter and Fcγ receptors. Arthritis Rheum 63:1446–1451. doi:10.1002/art.30274. 21538321

[37] Lood C, Gullstrand B, Truedsson L, Olin AI, Alm GV, Rönnblom L, Sturfelt G, Eloranta ML, Bengtsson AA. 2009. C1q inhibits immune complex-induced interferon-alpha production in plasmacytoid dendritic cells: a novel link between C1q deficiency and systemic lupus erythematosus pathogenesis. Arthritis Rheum 60:3081–3090. doi:10.1002/art.24852. 19790049

[38] Eloranta ML, Alm GV, Rönnblom L. 2013. Disease mechanisms in rheumatology–tools and pathways: plasmacytoid dendritic cells and their role in autoimmune rheumatic diseases. Arthritis Rheum 65:853–863. doi:10.1002/art.37821. 23280551

[39] Sturfelt G, Bengtsson A, Klint C, Nived O, Sjoholm A, Truedsson L. 2000. Novel roles of complement in systemic lupus erythematosus— hypothesis for a pathogenetic vicious circle. J Rheumatol 27:661–663. 10743804

[40] Lamb CA, Dooley HC, Tooze SA. 2013. Endocytosis and autophagy: shared machinery for degradation. Bioessays 35:34–45. doi:10.1002/bies.201200130. 23147242

[41] Guerra F, Bucci C. 2016. Multiple roles of the small GTPase Rab7. Cells 5:34. doi:10.3390/cells5030034. 27548222 PMC5040976

[42] Chua CE, Gan BQ, Tang BL. 2011. Involvement of members of the Rab family and related small GTPases in autophagosome formation and maturation. Cell Mol Life Sci 68:3349–3358. doi:10.1007/s00018-011-0748-9. 21687989 PMC11114630

[43] Agola JO, Jim PA, Ward HH, Basuray S, Wandinger-Ness A. 2011. Rab GTPases as regulators of endocytosis, targets of disease and therapeutic opportunities. Clin Genet 80:305–318. doi:10.1111/j.1399-0004.2011.01724.x. 21651512 PMC3187864

[44] Grosshans BL, Ortiz D, Novick P. 2006. Rabs and their effectors: achieving specificity in membrane traffic. Proc Natl Acad Sci USA 103:11821–11827. doi:10.1073/pnas.0601617103. 16882731 PMC1567661

[45] Poteryaev D, Datta S, Ackema K, Zerial M, Spang A. 2010. Identification of the switch in early-to-late endosome transition. Cell 141:497–508. doi:10.1016/j.cell.2010.03.011. 20434987

[46] Vitali C, Bombardieri S, Jonsson R, Moutsopoulos HM, Alexander EL, Carsons SE, Daniels TE, Fox PC, Fox RI, Kassan SS, Pillemer SR, Talal N, Weisman MH, European Study Group on Classification Criteria for Sjögren's Syndrome. 2002. Classification criteria for Sjogren’s syndrome: a revised version of the European criteria proposed by the American-European Consensus Group. Ann Rheum Dis 61:554–558. doi:10.1136/ard.61.6.554. 12006334 PMC1754137

[47] Aletaha D, Neogi T, Silman AJ, Funovits J, Felson DT, Bingham CO, Birnbaum NS, Burmester GR, Bykerk VP, Cohen MD, Combe B, Costenbader KH, Dougados M, Emery P, Ferraccioli G, Hazes JMW, Hobbs K, Huizinga TWJ, Kavanaugh A, Kay J, Kvien TK, Laing T, Mease P, Ménard HA, Moreland LW, Naden RL, Pincus T, Smolen JS, Stanislawska-Biernat E, Symmons D, Tak PP, Upchurch KS, Vencovsky J, Wolfe F, Hawker G. 2010. The 2010 rheumatoid arthritis classification criteria: an American College of Rheumatology/European League Against Rheumatism collaborative initiative. Ann Rheum Dis 69:1580–1588. doi:10.1136/ard.2010.138461. 20699241

[48] Hochberg MC. 1997. Updating the American College of Rheumatology revised criteria for the classification of systemic lupus erythematosus. Arthritis Rheum 40:1725. doi:10.1002/art.1780400928. 9324032

[49] Ganley Ian G, Wong PM, Gammoh N, Jiang X. 2011. Distinct autophagosomal-lysosomal fusion mechanism revealed by thapsigargin-induced autophagy arrest. Mol Cell 42:731–743. doi:10.1016/j.molcel.2011.04.024. 21700220 PMC3124681

