# Supplementary material for: CLEC18A Impairs Phagocytosis by Reducing FcγRIIA Expression and Arresting Autophagosome-Lysosome Fusion
Source: Microbiol Spectr. 2023 May 8;11(3):e02903-22. doi: 10.1128/spectrum.02903-22 (PMC10269929; doi:10.1128/spectrum.02903-22)
Supplement: Supplemental file 1 — Supplemental material. Download spectrum.02903-22-s0001.pdf, PDF file, 13.5 MB [file spectrum.02903-22-s0001.pdf]

Supplementary information

CLEC18A impairs phagocytosis by reducing FcγRIIA expression and arresting autophagosome–lysosome fusion

Tsai-Ling Liao[1,2,3*], Yi-Ming Chen[1-4], Kuo-Tung Tang[4], Ying-Ying Yang[5], Der-Yuan Chen[6-9], Tsung-Hsien Chan[1], Hui-Ju Tsai[1], Shie-Liang Hsieh[10-12*]

[1]Department of Medical Research, Taichung Veterans General Hospital, Taichung 407, Taiwan
[2]Ph.D. Program in Translational Medicine, National Chung Hsing University, Taichung 402, Taiwan
[3]Rong Hsing Research Center for Translational Medicine, National Chung Hsing University, Taichung 402, Taiwan
[4]Division of Allergy, Immunology and Rheumatology, Taichung Veterans General Hospital, Taichung 407, Taiwan
[5]Division of Gastroenterology and Hepatology, Taipei Veterans General Hospital, Taipei 112, Taiwan
[6]Rheumatology and Immunology Center, China Medical University Hospital, Taichung 404, Taiwan
[7]Translational Medicine Laboratory, Rheumatology and Immunology Center, China Medical University Hospital, Taichung 404, Taiwan
[8]College of Medicine, China Medical University, Taichung 404, Taiwan
[9]Institute of Medicine, Chung Shan Medical University Hospital, Taichung 402, Taiwan
[10]Genomics Research Center, Academia Sinica, Taipei 115, Taiwan
[11]Faculty of Medicine, National Yang Ming University, Taipei 112, Taiwan
[12]Immunology Research Center, National Health Research Institutes, Zhunan, Miaoli 35053, Taiwan

Corresponding author and address reprint requests:

Dr. Tsai-Ling Liao, Department of Medical Research, Taichung Veterans General Hospital, Taichung, Taiwan

Address: No.1650, Sec.4, Taiwan Boulevard, Xitun Dist., Taichung City 40705, Taiwan

Tel.: 886-4-23592525, extension 4020; Fax: 886-4-23592705
Email: tlliao@vghtc.gov.tw
*Co-corresponding author:
Professor Shie-Liang Hsieh, Genomics Research Center, Academia Sinica, Taipei, Taiwan
Address: No. 128, Section 2, Academia Road, Nankang, Taipei 11529, Taiwan
Tel.: 886-2-27871245; Fax: 886-2-27898811
Email: slhsieh@gate.sinica.edu.tw

Supplementary Materials

Reagent or Resource	Source	Identifier
Antibodies		
Mouse-anti-human 8-OHdG antibody	StressMarq Biosciences	SMC-15
Mouse anti-β-actin antibody (C4)	Santa Cruz	sc-47778
Mouse anti-CLEC18A antibody (3A9E6)	Sigma-Aldrich	MABS2260
Mouse anti-EEA1 antibody	BD Pharmingen	610456
Mouse anti-EGFR antibody	Santa Cruz	sc-120
Mouse anti-Hepatitis C Virus NS5a antibody (H26)	Abcam	ab13833
Mouse anti-FcγR2A (CD32) antibody (clone IV.3)	STEMCELL	#60012
Mouse anti-LAMP-1 antibody	BD Pharmingen	555798
Rabbit anti-Atg5 antibody	Cell Signaling Technology	#12994
Rabbit anti-CLEC18A Polyclonal antibody	proteintech	21013-1-AP
Rabbit anti-GM130 antibody (EP892Y)	Abcam	ab52649
Rabbit anti-LC3B antibodies	Cell Signaling Technology	#2775
Rabbit anti-p62 antibodies	Cell Signaling Technology	#8025
Rabbit anti-Rab5 antibody (C8B1)	Cell Signaling Technology	#3547
Rabbit anti-Rab7 antibody (D95F2)	Cell Signaling Technology	#9367T
Sheep anti-human TGN46 antibody	BIO-RAD	AHP500
Alexa Fluor® 647 Goat anti-mouse IgG (H+L)	Thermo Fisher Scientific	A21235
Alexa Fluor® 647 Goat anti-rabbit IgG (H+L)	Thermo Fisher Scientific	A21244
Anti-mouse IgG, HRP-linked Antibody	Cell Signaling Technology	#7076
Anti-rabbit IgG, HRP-linked antibody	Cell Signaling Technology	#7074
Chemicals and Assay kits		
3-methyladenine (3-MA)	Sigma-Aldrich	M9281
Ficoll-Paque Premium	GE Healthcare Biosciences	GE17-5442-02
CpG ODN2395	InvivoGen	tlrl-2395

Dextran Texas Red	Thermo Fisher Scientific	D1863
Diphenyleneiodonium chloride	Sigma-Aldrich	D2926
Phorbol myristate acetate	Sigma-Aldrich	P1585
poly (I:C)	InvivoGen	tlrl-pic
Resiquimod (R848)	InvivoGen	tlrl-r848
Recombinant human CLEC18A	MyBioSource	MBS1126756
QIAamp DNA Blood Mini Kit	QIAGEN	51106
RNeasy MinElute® Cleanup Kit	QIAGEN	74204
Trizol	Thermo Fisher Scientific	15596018
Dihydrorhodamine 123	Thermo Fisher Scientific	D23806
LysoTracker Green	Thermo Fisher Scientific	L7526
Human CLEC18A ELISA kit	CUSABIO	CSB-EL005521HU
Human IFNα ELISA kit	Thermo Fisher Scientific	LTMBMS216INST
Human CLEC18A TaqMan Gene Expression Assays	Thermo Fisher Scientific	Hs05310551_g1
Human FcγR2A TaqMan Gene Expression Assays	Thermo Fisher Scientific	Hs01013401_g1
Human FcγR2B TaqMan Gene Expression Assays	Thermo Fisher Scientific	Hs01634996_s1
Human FcγR3A TaqMan Gene Expression Assays	Thermo Fisher Scientific	Hs02388314_m1
Human FcγR3B TaqMan Gene Expression Assays	Thermo Fisher Scientific	Hs04334165_m1
On TARGETplus SMARTpool siCLEC18A	Dharmacon	L-018779-02-0005
Phagocytosis Assay Kit (IgG FITC)	Cayman	500290
OptiPrep	STEMCELL	07820
Rab7 (NM_004637) Human Tagged ORF Clone	OriGene	RC201776
Rab5 (NM_004162) Human Tagged ORF Clone	OriGene	RC203873
Universal IP/Co-IP Toolkit	Abbkine	KTD104-EN

Supplementary Methods

Serological and virological evaluation for HCV infection

HCV infection was diagnosed using commercial third-generation ELISA kits (J. Mitra & Co. Pvt. Ltd., New Delhi, India) to detect anti-HCV antibodies, and HCV viremia by the polymerase chain reaction of anti-HCV antibodies was detected. The serum HCV viral loads were quantified using the Roche COBAS TaqMan HCV Test (Roche Diagnostics, Switzerland) according to the manufacturer's instructions. The viral load was expressed as log10 of the detected values for analysis.

HCV infection system

The pJC1 plasmid contains a chimera genome of HCV J6CF/JFH1, was constructed as previously described [1]. Full-length JC1 genomic RNA was produced by *in vitro* transcription of pJC1, and electroporated into Huh7.5 cells, and incubated for 24 h. JC1 viral particles were then collected from the cell culture supernatant at 3 day after transfection for further expansion. HCV infection was based on a method previously described [2], with minor modifications. Briefly, Huh-7.5 cells were seeded at a density of 1×10^6 cells. After 24 h, cells were infected with HCV JC1 strain at MOI of 0.1 for 3 h, washed with PBS, and then added to complete medium and cultured at 37°C for 72h.

Autophagosome maturation

THP-1 cell-derived macrophages were grown on coverslips at a concentration of 1 × 10^5 cells/well. CLEC18A (40 ng/ml) or BafA1 (100 nM) was added to the cells for 24 h before infection. The cells were infected with Texas red-labeled *M. bovis* BCG at an MOI of 10 for 1 h, washed three times with PBS to remove unbound mycobacteria, and incubated with LysoTracker Green (Thermo Fisher Scientific, Fremont, CA, USA) for the final 1 h. The cells were fixed in 4% paraformaldehyde for 10 min at room temperature. Coverslips were mounted onto glass slides with a mounting medium (Thermo Fisher Scientific), and the images were recorded on an Olympus FV1000 laser scanning confocal microscope.

Immunoprecipitation

The immunoprecipitation assay was performed using Universal IP/Co-IP Toolkit (Abbkine, China) according to the manufacturer's instructions. The 293T cells were transfected in 6-well plates by use of Lipofectamine 3000 (Thermo Fisher Scientific). Approximately 24 h after transfection, cells were washed with PBS and lysed with lysis buffer. After centrifugation at 12,000 × g at 4°C for 15 min, the protein was collected and quantified. Thirty micrograms of total proteins collected from each sample incubated with 0.75μg of the indicated antibodies or rabbit IgG (negative control) for overnight at 4°C. The immunoprecipitated proteins were analyzed by

using immunoblotting with the indicated antibodies.

Reference

1. Pietschmann T, Kaul A, Koutsoudakis G, Shavinskaya A, Kallis S, et al. Construction and characterization of infectious intragenotypic and intergenotypic hepatitis C virus chimeras. Proc Natl Acad Sci U S A 2006; 103: 7408–7413.
2. Chao TC, Su WC, Huang JY, Chen YC, Jeng KS, Wang HD, Lai MMC. Proline-serine-threonine phosphatase-interacting protein 2 (PSTPIP2), a host membrane-deforming protein, is critical for membranous web formation in hepatitis C virus replication. J Virol. 2012; 86: 1739–1749.

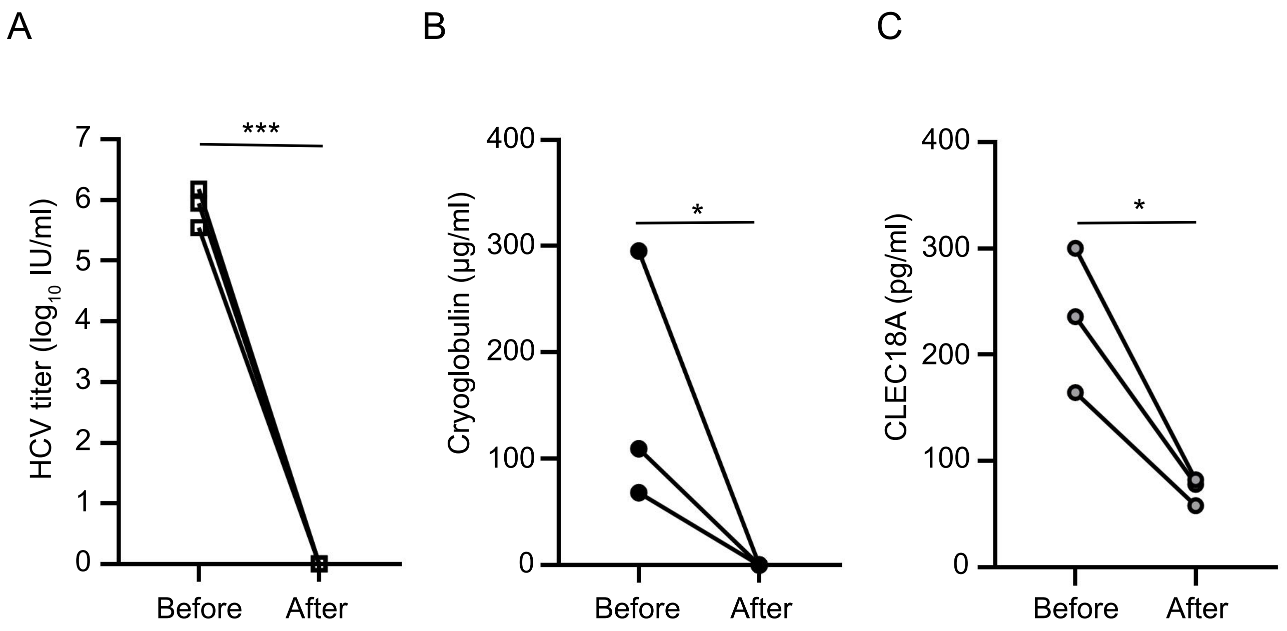

Figure S1 The dynamic of (A) the HCV titer, (B) cryoglobulin, and (C) CLEC18A expression in sera of HCV-MC patients receiving direct-acting antiviral (DAA) drugs therapy. The data are presented as the mean ± SD. $*P < 0.05$, $***P < 0.005$.

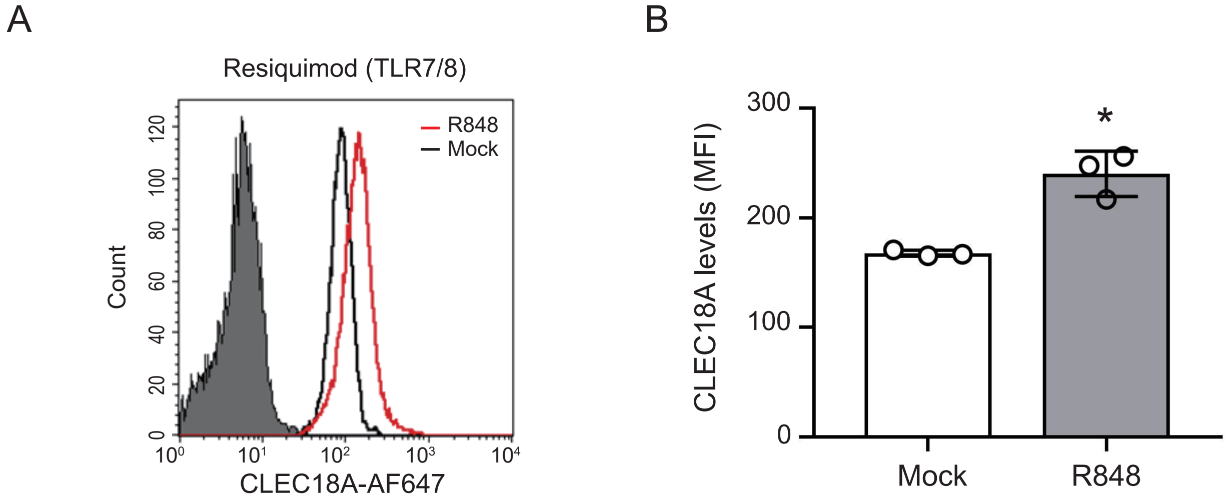

Figure S2 Increased CLEC18A levels in human neutrophils after TLR7/8 ligand treatment.
(A) Human neutrophils were treated with indicated resiquimod (R848) for 24h, CLEC18A expression was analyzed by flow cytometry assay and (B) quantified. All experiments were performed in triplicate, and data are presented as the mean ± SD. *$P < 0.05$.

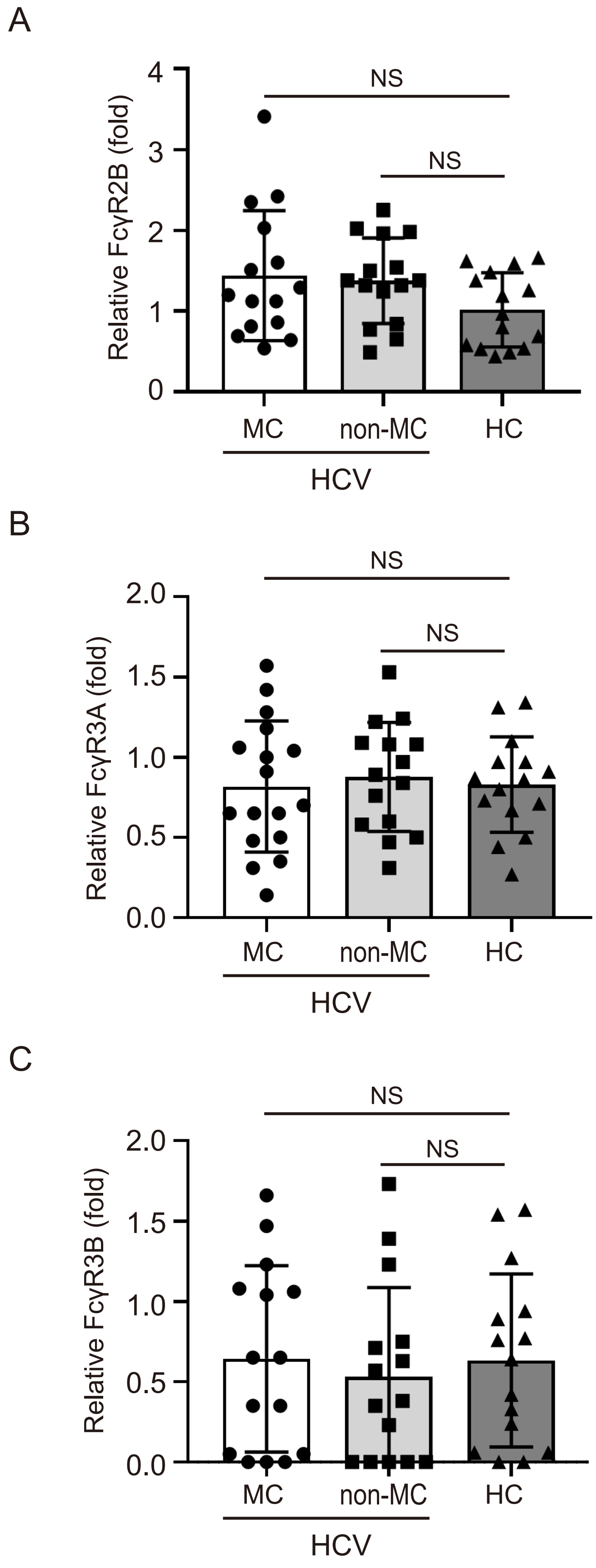

Figure S3 The levels of (A)FcγRIIB, (B)FcγRIIIA, and (C)FcγRIIIB in neutrophils of patients with or without HCV-associated mixed cryoglobulinemia (MC), and healthy control (HC) were measured by quantitative reverse transcription PCR (QRT-PCR). All experiments were performed in triplicate, and data are presented as the mean ± SD. NS, no significant.

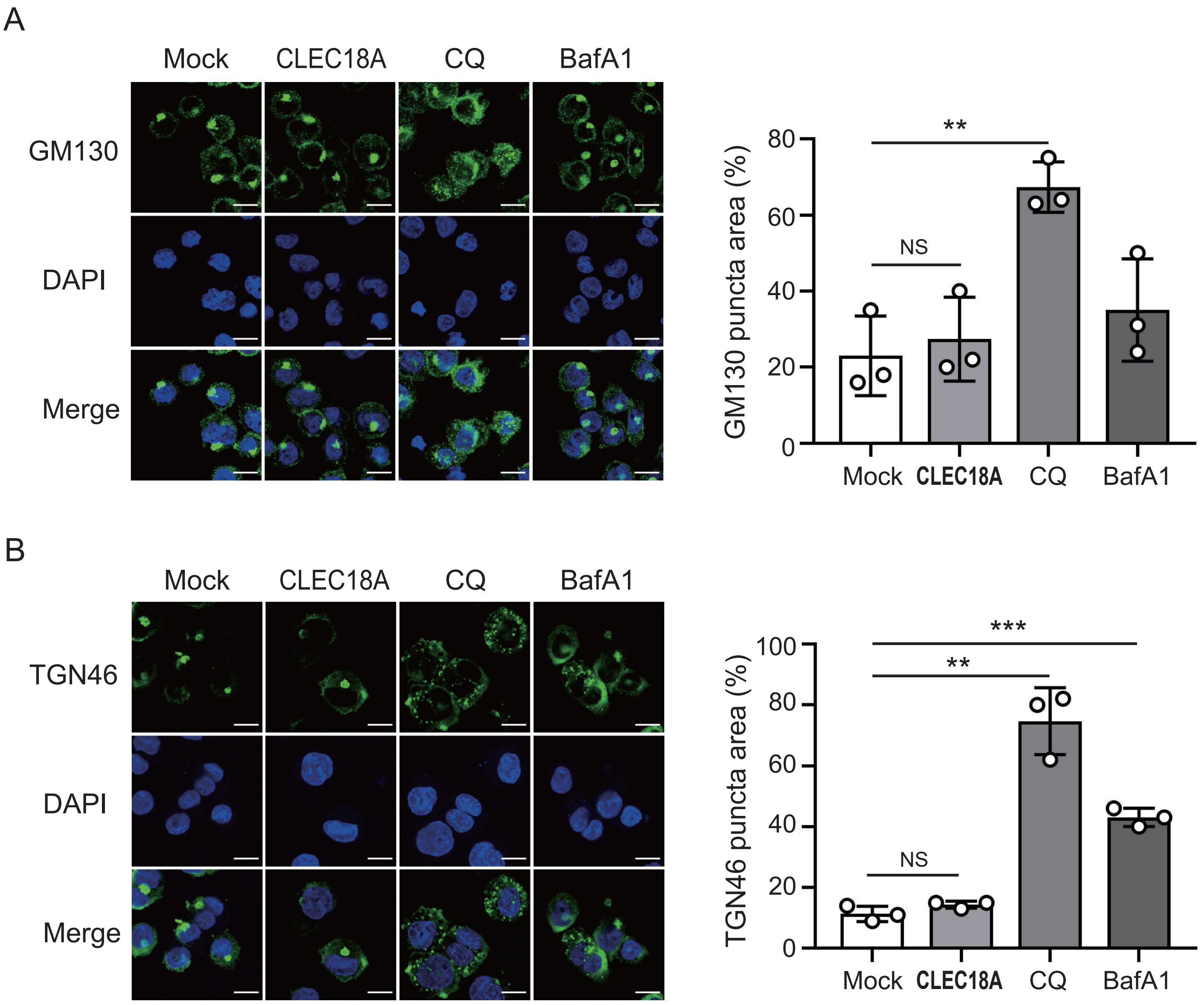

Figure S4 CLEC18A has no significant impact on the Golgi complex in phagocytosis. THP-1 cell-derived macrophages were treated with CLEC18A (40 ng/ml), chloroquine (CQ, 100μM) , or bafilomycin A1 (100 nM) for 24 h. The cells were stained with antibodies against (A) cis-Golgi (GM130),or (B) trans-Golgi (TGN46) marker proteins. The number of puncta per cell were detected by confocal microscopy and quantified. All the experiments were performed in triplicate, and the data is presented as the mean±SD. The scale bar in the IFA image represents 10 μm. NS, no significant. **$P < 0.01$, ***$P < 0.005$.

A

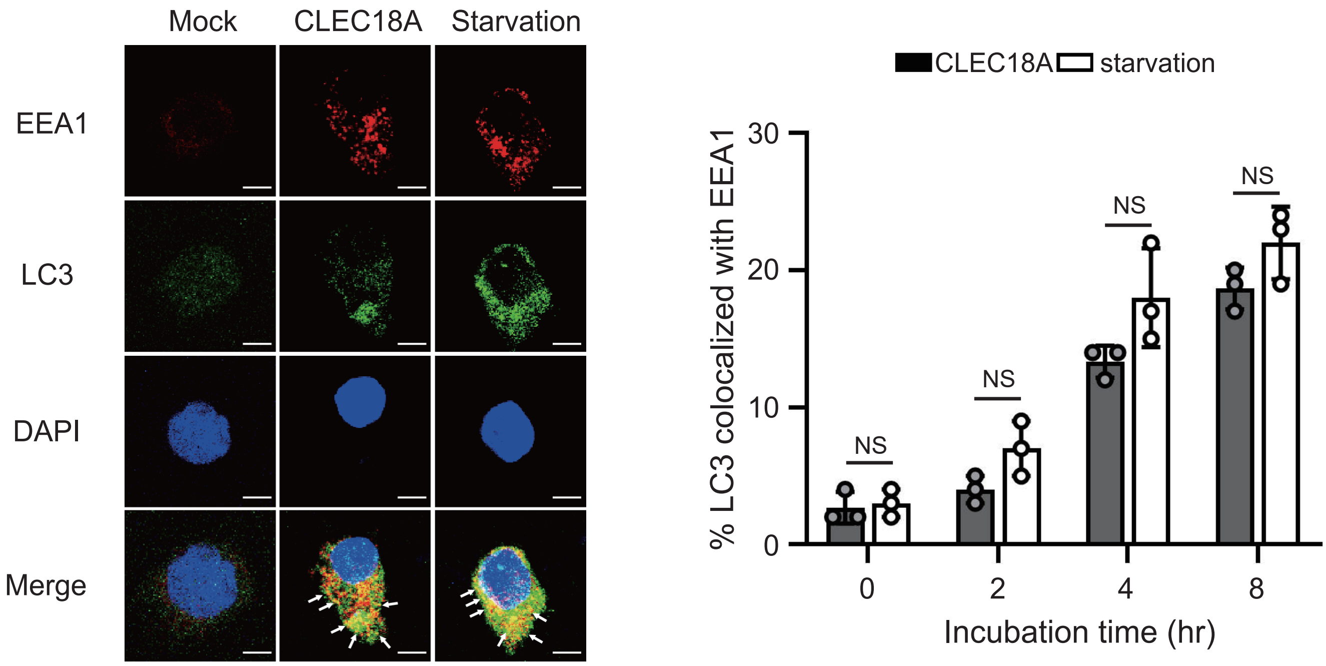

B

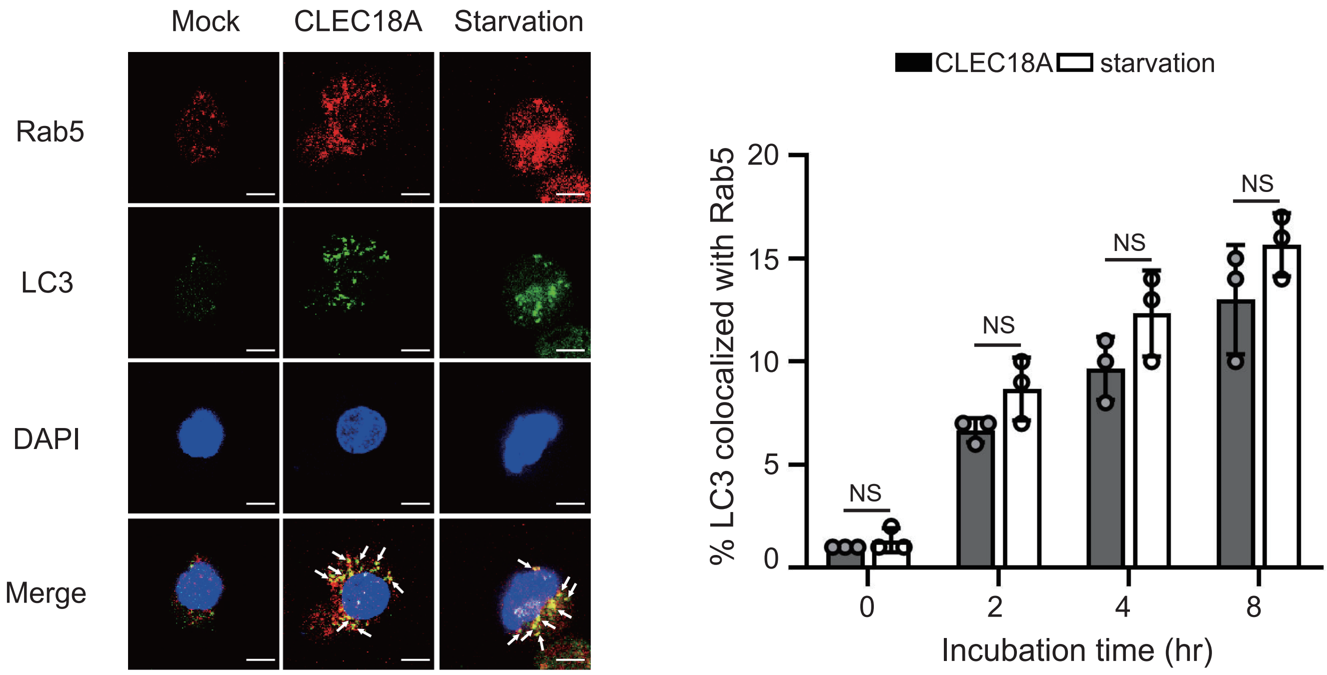

Figure S5 THP-1 cell-derived macrophages were starved in amino acid–free media or treated with CLEC18A (40 ng/ml) for the indicated time. The cells were stained with antibodies against LC3 (green) and (A) EEA1(red) or (B) Rab5 (red), respectively. The co-localization of EEA1 or Rab5 and LC3 puncta were detected via confocal microscopy (left panel, the represented image: 4 h) and calculated (right panel). All the experiments were performed in triplicate, and the data is presented as the mean±SD. The scale bar in the IFA image represents 10 μm. NS, no significant.

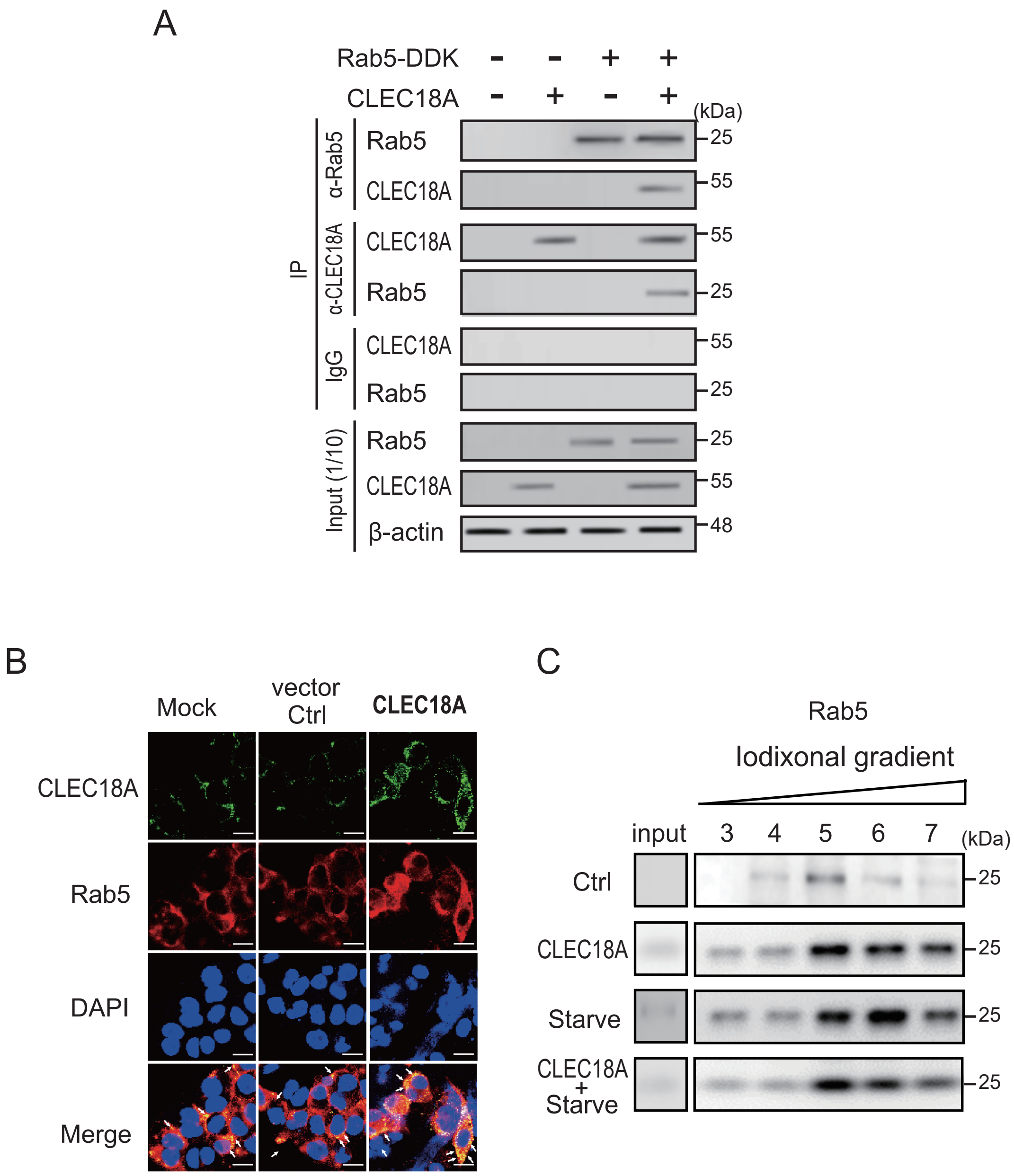

Figure S6 CLEC18A specifically interacts with Rab5. (A) 293T cells were mock transfected, or transfected with pCMV-Myc-DDK tagged Rab5 and pCMV-CLEC18A. Twenty-four hours post-transfection, the cell lysates were subjected to reciprocal immunoprecipitation assays with an anti-Rab5, anti-CLEC18A antibodies, or rabbit IgG (negative control), respectively. Immunoprecipitated proteins were separated and then visualized by immunoblotting with an anti-Rab5 antibody or an anti-CLEC18A antibody. (B) THP-1–derived macrophages were mock transfected, or transfected with pCMV-CLEC18A or vector control. 24 hours post-transfection, the co-localization of CLEC18A and Rab5 was detected via confocal microscopy. (C) THP-1–derived macrophages were treated with the indicated reagent for 24 h. The cell homogenates were subjected to iodixanol density gradient centrifugation (10%–50% gradient) and fractionated. The expression of Rab5 was detected by using immunoblotting. All the experiments were performed in triplicate, and the data are presented as the mean ± SD. The scale bar in the IFA image represents 10 μm.

A

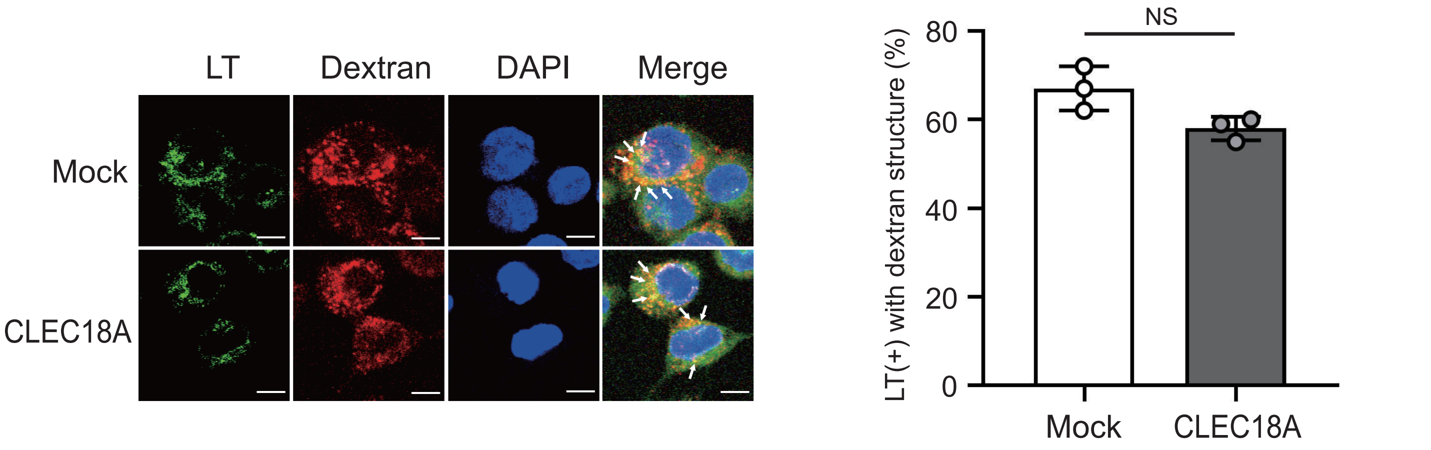

B

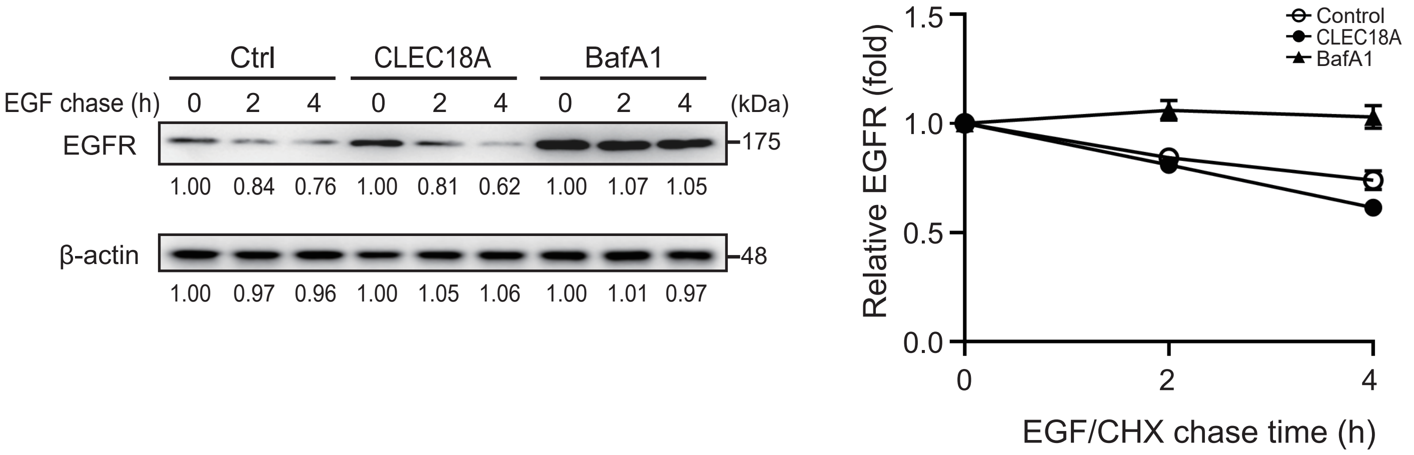

Figure S7 CLEC18A does not inhibit endocytosis. (A) THP-1 cell-derived macrophages were incubated in complete media with or without CLEC18A (40 ng/ml) for 24 hr, then added with Texas red-labeled dextran for 2 hr and stained with LysoTracker Green (LT, 2 μM).The co-localization of dextran with LT was detected by confocal microscopy and quantified. (B) THP-1 cell-derived macrophages were incubated in serum-free RPMI for 3 h, and then 40 ng/ml CLEC18A (or PBS control) was added for an additional 2 h. The media was then replaced with serum-free RPMI containing 40 ng/ml EGF and 20 mg/ml cycloheximide (CHX), with or without CLEC18A (40 ng/ml) or bafilomycin A1 (BafA1, 100 nM) for the indicated times. The cells were lysed and subjected to immunoblotting analysis. All the experiments were performed in triplicate, and the data is presented as the mean±SD. The scale bar in the IFA image represents 10 μm. EGFR, epidermal growth factor receptor. NS, no significant.

Supplementary Figure S8 Densitometric analysis of immunoblot results presented in this study. Immunoblots were quantitated by densitometric analysis using ImageJ software and normalized to β-actin. Numbers below each lane are relative fold of the control level of a specific protein in mock-treated cells. All results were obtained in three independent experiments, and the data is presented as the mean±SD. *P<0.05, ** P<0.01, *** P<0.005.

Fig. 1J

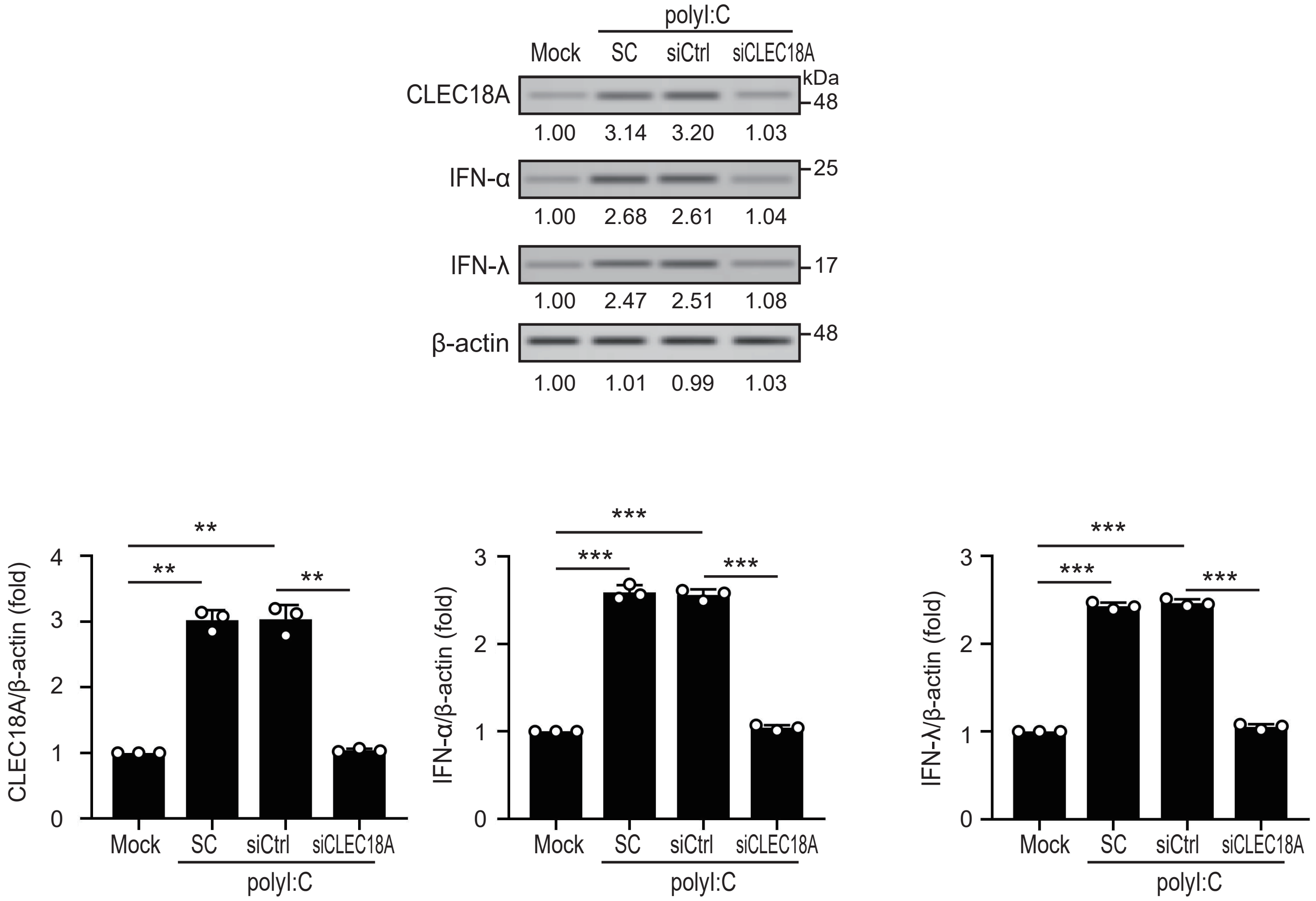
polyI:C
Mock
SC
siCtrl
siCLEC18A
kDa
CLEC18A
48
1.00 3.14 3.20 1.03
IFN-α
25
1.00 2.68 2.61 1.04
IFN-λ
17
1.00 2.47 2.51 1.08
β-actin
48
1.00 1.01 0.99 1.03
CLEC18A/β-actin (fold)
IFN-α/β-actin (fold)
IFN-λ/β-actin (fold)
**
***
0
1
2
3
4
Mock
SC
siCtrl
siCLEC18A
polyI:C

Fig. 4E

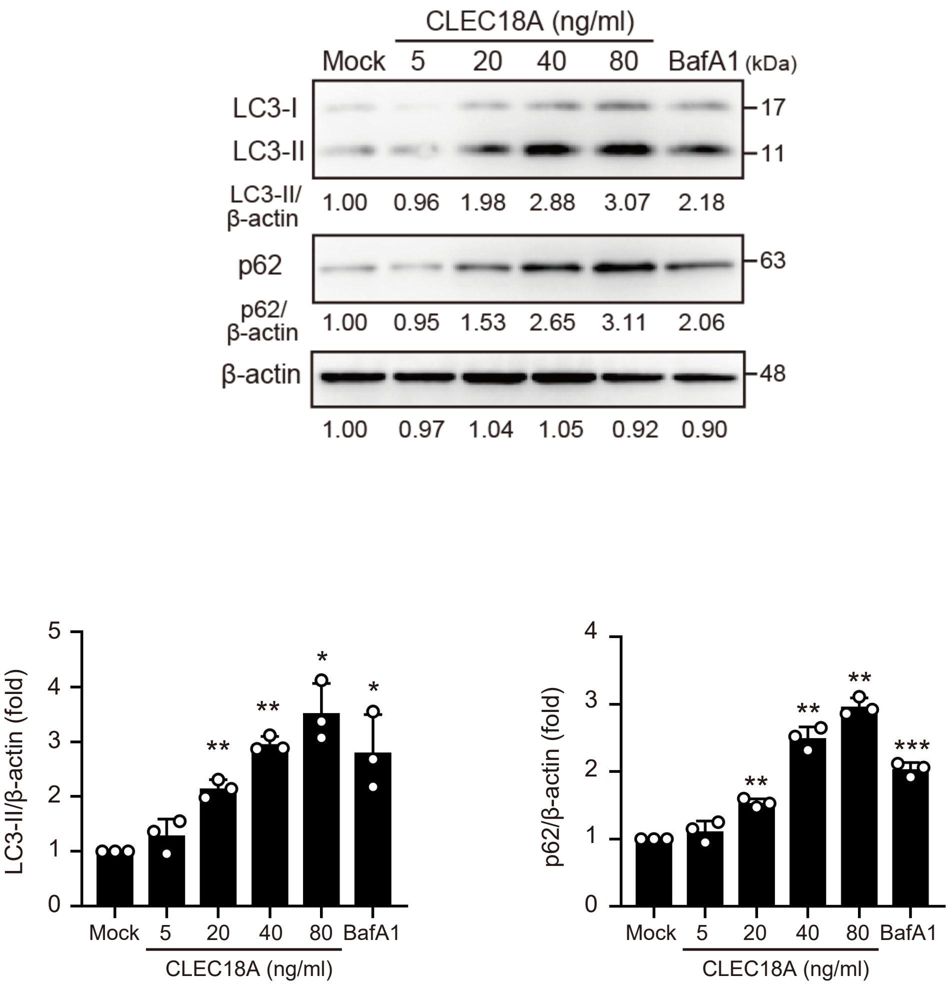

CLEC18A (ng/ml)
Mock 5 20 40 80 BafA1 (kDa)
LC3-I
LC3-II
17
11
LC3-II/ β-actin 1.00 0.96 1.98 2.88 3.07 2.18
p62
63
p62/ β-actin 1.00 0.95 1.53 2.65 3.11 2.06
β-actin
48
1.00 0.97 1.04 1.05 0.92 0.90
LC3-II/β-actin (fold)
p62/β-actin (fold)
Mock 5 20 40 80 BafA1
CLEC18A (ng/ml)

Fig. 4G

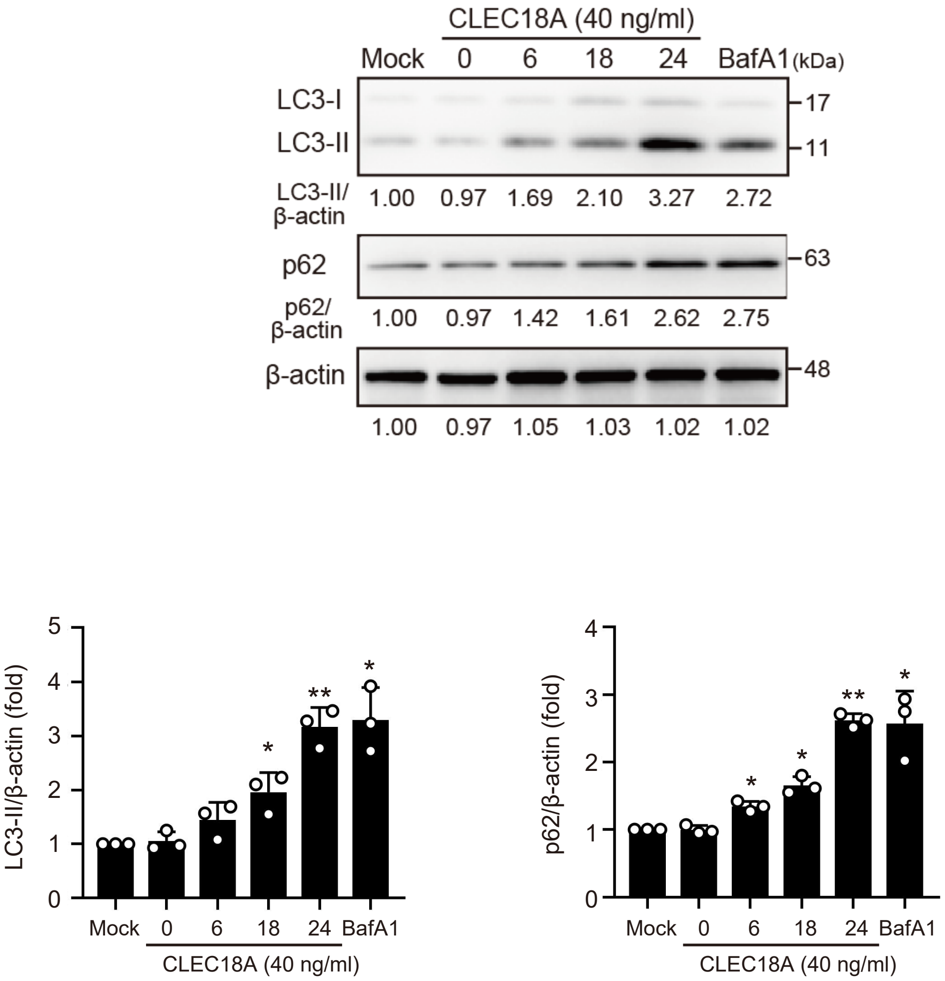
CLEC18A (40 ng/ml)
Mock 0 6 18 24 BafA1 (kDa)
LC3-I
LC3-II
17
11
LC3-II/β-actin 1.00 0.97 1.69 2.10 3.27 2.72
p62
63
p62/β-actin 1.00 0.97 1.42 1.61 2.62 2.75
β-actin
48
1.00 0.97 1.05 1.03 1.02 1.02
LC3-II/β-actin (fold)
0 1 2 3 4 5
*
**
*
Mock 0 6 18 24 BafA1
CLEC18A (40 ng/ml)
p62/β-actin (fold)
0 1 2 3 4
*
*
**
*
Mock 0 6 18 24 BafA1
CLEC18A (40 ng/ml)

Fig. 6B

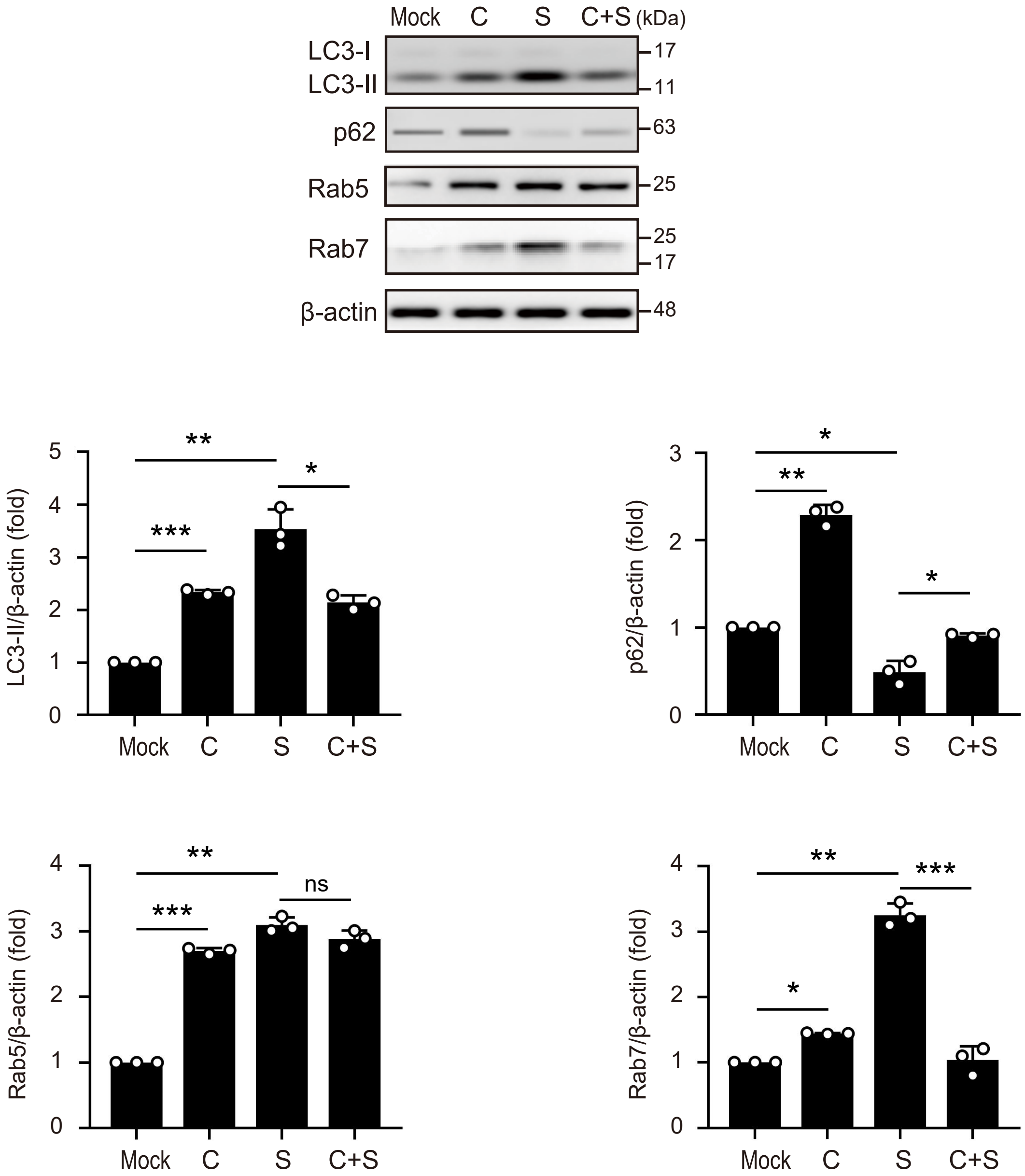
Mock
C
S
C+S
(kDa)
LC3-I
LC3-II
p62
Rab5
Rab7
β-actin
17
11
63
25
25
17
48
LC3-II/β-actin (fold)
p62/β-actin (fold)
Rab5/β-actin (fold)
Rab7/β-actin (fold)
**
***
*
ns
